# Age-dependent topoisomerase I depletion alters recruitment of rDNA silencing complexes

**DOI:** 10.1016/j.jbc.2025.111062

**Published:** 2025-12-17

**Authors:** Lindsey N. Power, Natalia Zawrotna, Manikarna Dinda, Abigail E. Weir, B. Bishal Paudel, Oshil Ghimire, Karolina Kisiel, Christopher T. Letai, Kevin A. Janes, Jeffrey S. Smith

**Affiliations:** 1 Department of Biochemistry and Molecular Genetics, University of Virginia School of Medicine, Charlottesville, Virginia, USA; 2 Department of Medical Chemistry, Medical University of Gdansk, Gdansk, Poland; 3 Division of Hematology & Oncology, Department of Medicine, Charlottesville, Virginia, USA; 4 Department of Biomedical Engineering, University of Virginia School of Medicine, Charlottesville, Virginia, USA; 5 College of Arts and Sciences, University of Virginia, Charlottesville, Virginia, USA; 6 College of Health Science, Physician Assistant Program Midwestern University, Downers Grove, Illinois, USA

**Keywords:** proteomics, replicative lifespan, aging, topoisomerase I, rDNA, yeast

## Abstract

Genomic instability and loss of proteostasis are two of the primary Hallmarks of Aging. Although these hallmarks are well-defined in the literature, the mechanisms that drive genomic instability and loss of proteostasis as cells age are still incompletely understood. Using budding yeast replicative lifespan as a model for aging in actively dividing cells, we identify nuclear proteins that are depleted in the earliest stages of aging. We find that many age-depleted proteins are involved in ribosome biogenesis, specifically in ribosome processing, or in maintenance of chromatin stability. We focus on topoisomerase I (Top1) as a novel age-depleted nuclear protein and determine that its depletion in the early stages of aging is not a result of transcriptional changes or changes in protein turnover. Despite the stark depletion of Top1 in early aging, we find that rescue of this age-dependent depletion is harmful to replicative lifespan. When overexpressed, Top1 disrupts the stoichiometry of the RENT complex by pulling Sir2 away from the ribosomal DNA (rDNA), a phenotype that is further enhanced when the overexpressed Top1 is catalytically dead. Loss of Sir2 from the rDNA *via* the overexpression of catalytically dead Top1 decreases RNA Pol II silencing of a reporter gene inside or adjacent to the rDNA, consistent with the lifespan defect. Finally, we show that the catalytic activity of Top1 plays an important role in the establishment of rDNA silencing, raising the possibility that rDNA secondary structure/DNA topology is important for RNA Pol I–dependent spreading of silent chromatin across the rDNA locus.

Aging across eukaryotes involves a series of declining cellular functions known commonly as the “Hallmarks of Aging.” The molecular pathways involved in some of these hallmarks, such as genome instability and loss of proteostasis, are highly conserved from yeast to humans (1, 2). Due to this high level of conservation, replicative aging in *Saccharomyces cerevisiae* (budding yeast) has long been an established model for studying the molecular mechanisms of aging in dividing cells since the discovery that yeast has a finite lifespan (2). The replicative lifespan (RLS) of budding yeast is quantified as the number of times a mother cell buds before death (2). Importantly, daughter cells bud asymmetrically from the aging mother cell and have full replicative potential, so RLS is considered a model for aging in dividing cells of higher eukaryotes, such as stem cells (3). A notable example of an aging-relevant discovery from yeast was the identification of Sir2 and other sirtuin family members as NAD^+^-dependent histone/protein deacetylases (HDACs) (4, 5, 6). Sir2 is the catalytic subunit of two HDAC complexes that function in heterochromatic gene silencing (7, 8). The first Sir2 HDAC complex, known as the SIR complex, consists of Sir2, Sir3, and Sir4, and is responsible for the establishment and maintenance of silencing at telomeres and the silent mating type loci (*HML* and *HMR*) (9, 10, 11). The second Sir2 HDAC complex is known as REgulator of Nucleolar silencing and Telophase exit (RENT) and consists of Sir2, Net1, and Cdc14 subunits (12, 13). RENT is recruited to the IGS1 and IGS2 intergenic spacers of the ribosomal DNA (rDNA) locus where it silences RNA Pol II–mediated transcription of several long non-coding RNAs (11, 14, 15, 16), a function required to maintain stability of the tandem array (17, 18). Importantly, recruitment of the RENT complex to the rDNA to induce silencing is partially dependent on active RNA Pol I transcription of the 35S rRNA gene (19).

The rDNA array is a key modulator of RLS. Moderate overexpression of Sir2 enhances rDNA silencing and extends RLS (20, 21). Furthermore, rDNA tandem array stability and array size have direct effects on RLS. For example, yeast mother cells accumulate extrachromosomal rDNA circles (ERCs) as they age due to unequal crossing over during DNA double-strand break repair at the Fob1-mediated replication fork block (22, 23). Destabilizing the array by deleting *SIR2* increases the number of ERCs and causes premature aging (20), but this increase in ERCs/reduced lifespan is partially rescued by deletion of *FOB1* (24), which suppresses ERC production. Additionally, yeast RLS correlates directly with rDNA copy number, as strains with greater rDNA copy number in the tandem array tend to have better longevity (25). Because rDNA stability is such an important modulator of lifespan, maintenance of rDNA regulatory and structural complexes such as RENT and others becomes critical as cells age.

Given that overall translational efficiency decreases globally in aged yeast cells (26), many important proteins may decline in abundance during replicative aging (26). A decline in certain protein levels can alter stoichiometry of multisubunit protein complexes and have negative consequences for RLS. For example, altered stoichiometry of nuclear pore complexes has been linked to replicative aging (27). Additionally, the dysregulation of protein level homeostasis in aging yeast has been linked to genome instability, as cells with increased rDNA instability undergo aggregation of important rRNA-binding proteins (28). Key rDNA silencing proteins like Sir2 are limiting within the cell, giving rise to competition between the rDNA locus and other regions of the genome where these proteins are functioning, such as telomeres (21). Potentially exacerbating this competition, Sir2 protein levels are significantly reduced in replicative aging yeast cells (29).

It has also been demonstrated that the cohesin subunits Mcd1 and Scc1 are depleted during replicative aging and that forced depletion of Mcd1 shortens RLS (30, 31). Mcd1 is depleted from the rDNA locus at the early stages of aging and redistributed to centromeres (30), most likely to retain the integrity of mitotic chromosome segregation. During the later stages of aging, any remaining Mcd1 is also depleted from centromeres, correlating with development of a general chromosome instability phenotype (30). Overexpression of *MCD1* from a doxycycline inducible promoter is sufficient to stabilize the rDNA and extend RLS (30), similar to the effect of increasing Sir2 expression (20). These studies led us to ask what other nuclear proteins are depleted during the early stages of replicative aging and thus potentially important in maintaining rDNA stability.

Using a mini-chemostat aging device (MAD) system (32), we performed a proteomic screen for nuclear proteins that significantly change in abundance during the early stages of replicative aging. We identified several important chromatin stability factors such as topoisomerases, helicases, and remodelers, as well as ribosome biogenesis factors that were significantly reduced. Focusing on topoisomerase I (Top1), due to its known role in maintaining chromatin stability, we performed rescue experiments to determine how Top1 re-expression in the early stages of aging, or overexpression in log-phase yeast, influences RLS and rDNA silencing, respectively. Here, we demonstrate the importance of Top1 homeostasis in maintaining RENT complex enrichment at the rDNA, and report both structural and enzymatic roles for Top1 in establishing rDNA silencing.

## Results

### Nuclear protein levels change significantly in the early stages of replicative aging

**Figure 1:**
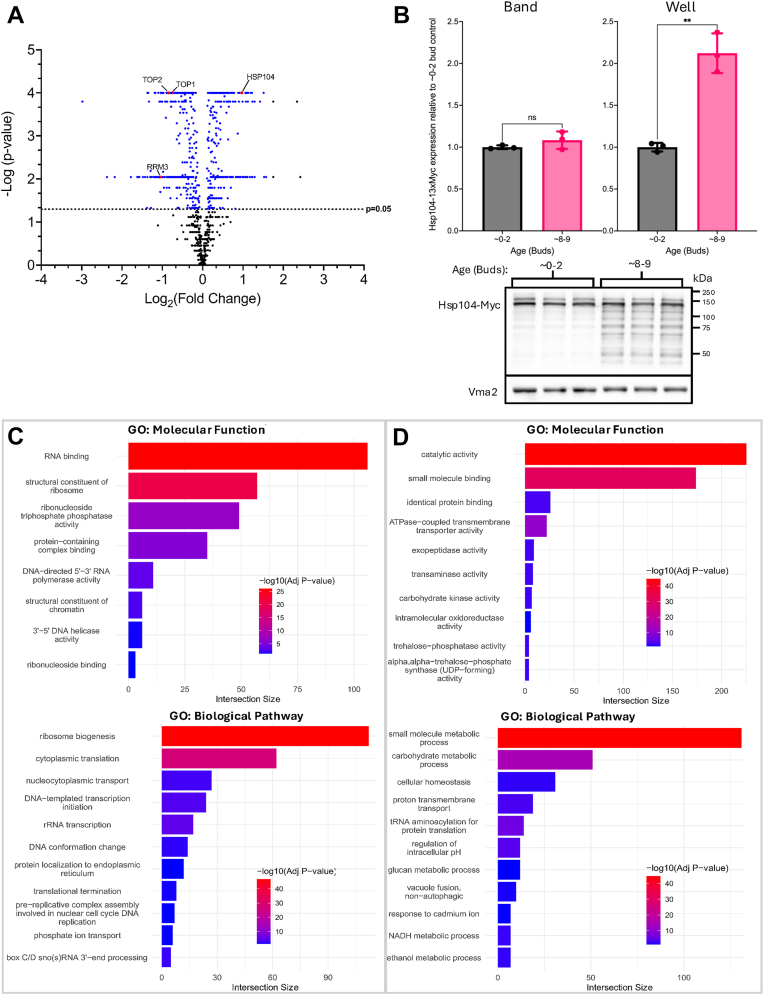
**Nuclear protein levels change in early replicative aging**. *A*, −log fold change in protein expression for nuclei isolated from moderately aged (∼6–7 buds) yeast (BY4743) relative to young (∼0–2 buds) controls obtained using TMT-mass spectrometry (n = 5 biological replicates, −log10 *p* values comparing change between old and young cells were calculated for each protein by ANOVA with corrections for multiple comparisons). *B*, Hsp104-13xMyc protein expression in whole cell extracts from young and aged (∼8–9 generations) MD188 cells (two-tailed student’s *t*-tests, *p* = 0.2460 and ∗∗*p* = 0.0013, n = 3 biological replicates). The loading control Vma2 does not significantly change during replicative aging (78). *C*, GO term enrichment for age-depleted proteins using the molecular function (*top*) or biological pathway (*bottom*) models using g-Profiler (34). *D*, significant GO term enrichment for upregulated proteins. TMT, tandem mass tag.

Changes in intracellular protein levels of very old yeast cells (>25 generations) are influenced by a decrease in global translational efficiency (26), but the changes that occur in the early stages of replicative aging are not yet known. To better understand changes in nuclear protein landscape during the initial stages of aging prior to cells senescing, we performed a proteomic screen comparing isolated nuclei from young (∼0–2 generations) *versus* moderately aged (∼6–7 generations) yeast cells (diploid lab strain BY4743) that were isolated from MADs (see Experimental Procedures). Following isolation of nuclei, we verified nuclear enrichment by 4′,6-diamidino-2-phenylindole staining under a fluorescence microscope (Fig. S1*A*) and by confirming high levels of histone acetyltransferase (HAT) activity relative to purified HAT1 as a positive control (Fig. S1*B*). We then performed tandem mass tag (TMT)-mass spectrometry on the purified nuclei and performed differential analysis on protein levels in the old nuclei compared to the young nuclei (Fig. 1*A*). Roughly 70% of the ∼1000 proteins identified in the screen were unchanged in the aged samples, while 159 were significantly upregulated and 248 significantly downregulated in the aged samples (Fig. 1*A*, Table S5). To verify results from the proteomic screen, we first chose one of the significantly upregulated proteins, Hsp104, to tag at the C terminus with the 13xMyc epitope. Hsp104 was chosen because foci of this chaperone protein accumulate in aged cells as it interacts with damaged and misfolded proteins (33). We aged the *HSP104*-13xMyc–tagged strain (MD188) for 36 h (∼8 generations) on the mini-chemostat system and performed Western blots on whole cell protein extracts (Fig. 1*B*). Comparison of aged cell Hsp104-Myc protein expression to young cell controls showed a significant increase in total Hsp104 expression, although the full-length protein remained unchanged. This result suggested that the 13xMyc epitope tag was likely being processed in the older cells at the whole cell level as Hsp104 protein expression increases and foci accumulate (Fig. 1*B*). Next, gene enrichment analysis was performed for the significantly depleted (Fig. 1*C*) or significantly increased (Fig. 1*D*) proteins using the molecular function and biological pathway gene ontology term activity models from g:Profiler (34). Among the significantly decreased proteins that were purified with the nuclei, many were involved in ribosome biogenesis, such as RNA polymerase I and III subunits and the rRNA processing machinery. We also note significant reduction of ribosomal subunits, which are assembled into ribosomes within the nucleolus. Also of particular interest was downregulation of proteins with roles in the maintenance of chromatin structure and stability, including the core histones and histone variants. Histones are reduced in the later stages of aging (35), and we have now confirmed this with earlier stages of aging, thus further validating results from the proteomic screen. Proteins that were significantly increased with age include many involved in the maintenance of cellular homeostasis, including metabolic processes such as carbohydrate, glucan, NADH, and ethanol metabolism (Fig. 1*D*).

### Top1 depletion in early aging is not from decreased transcription or increased protein turnover

**Figure 2:**
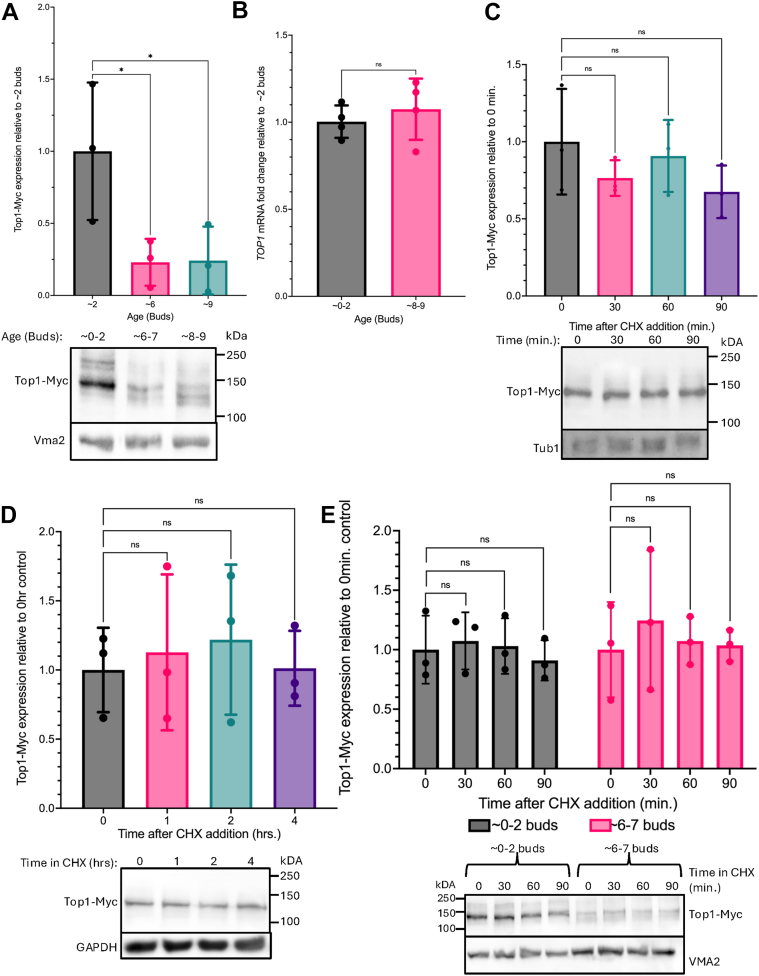
**Top1 is depleted at the protein level during replicative aging**. *A*, quantitation of Top1-13xMyc depletion from LP128 cells aged to ∼6 or 9 generations compared to young controls (∼0–2 generations). (One-way ANOVA with Dunnett’s test for multiple comparisons, ∗*p* = 0.0454 and ∗*p* = 0.0482, n = 3 biological replicates) *B*, qRT-PCR results showing mRNA fold change of *TOP1* after ∼8 to 9 generations relative to their young control (∼0–2 generations) (two-tailed *t* test, *p* = 0.4988 n = 4 biological replicates). *C*, Top1-Myc expression after 0, 30, 60, and 90 min chase in 250 μg/ml CHX relative to the 0 min control. *D*, Top1-Myc expression after 0, 1, 2, and 4 h chase in 250 μg/ml CHX relative to the 0 h control. *E*, Top1-Myc expression in young (∼0–2 buds) and moderately aged (∼6–7 buds) after 0,30,60, and 90 min incubation in 250 ug/ml CHX relative to the 0 min control. All CHX assays were analyzed by one-way ANOVAs with Dunnett’s test for multiple comparisons, adjusted *p* values were all ns (*p* > 0.05), n = 3 biological replicates. Exact *p* values are listed in Table S4. CHX, cycloheximide; Top 1, topoisomerase 1.

Previous work from our lab and others demonstrated age-associated depletion and redistribution of important rDNA stabilizing proteins such as Sir2 and the Mcd1 subunit of cohesin (30, 31, 36), both of which can extend RLS upon overexpression (30). We thus followed up on age-depleted proteins involved in chromatin stability, as we predicted the depletion of these proteins in early aging would negatively impact RLS. We focused on Top1, as it has important dual roles in (i) relieving supercoiling associated with DNA replication and genome-wide transcription and (ii) recruitment of Sir2 to the rDNA locus (37, 38, 39). Using Western blotting, we first verified that Top1-Myc (strain LP128) was significantly depleted in mother cells replicative aged for either ∼6 (18 h) or ∼9 (36 h) generations in the mini-chemostats (Fig. 2*A*). Top1-myc was strongly depleted to ∼20% within 6 generations, thus validating proteomic results and also confirming that the 13x-myc tag was not artificially stabilizing the fusion protein during aging.

We next verified that the 13xMyc epitope tag was not affecting the rDNA silencing function of Top1. Since deletion of *top1* causes a silencing defect in yeast due to a loss of Sir2 from the rDNA locus (15, 39), we performed quantitative RT-PCR (qRT-PCR) for the IGS1 lncRNA that is transcribed from the rDNA when silencing is defective (Fig. S2). Relative to an untagged WT control BY4741, the IGS1 lncRNA expression level from the Top1-Myc–tagged strain was not significantly changed, while IGS1 lncRNA expression from a *sir2Δ* control strain was significantly increased relative to the WT thus demonstrating that the Myc-tag epitope does not significantly impact Top1 rDNA silencing activity.

We next asked if age-dependent Top1 protein depletion was due to decreased transcription of the *TOP1* gene, isolating RNA from young and moderately aged cells for qRT-PCR assays. *TOP1* mRNA expression levels were unchanged in aged cells (Fig. 2*B*), indicating that decreased transcription or mRNA stability was not the cause of the age-induced protein depletion. We also hypothesized that there could be faster Top1 protein turnover induced by aging. To test this idea, a series of cycloheximide (CHX) chase assays was performed. We first verified that a final concentration of 250 μg/ml CHX, commonly used for such experiments in yeast, was sufficient to arrest cell growth and confirmed the compound was effectively inhibiting translation (Fig. S3*A*). As positive control, a 4-h CHX assay was performed on a temperature sensitive Cdc13-13xMyc protein with a short half-life of less than 1 h (Fig. S3*B*) (40). The half-life of Top1 has not been published, so we initially ran a CHX chase experiment on log-phase cells and did not observe any significant turnover of Top1-Myc protein after 90 min of CHX incubation (Fig. 2*C*). We therefore increased the maximum incubation time of the cells in CHX to 4 h and again did not observe any significant changes in Top1-Myc levels (Fig. 2*D*), indicating that Top1 is a stable protein in young exponentially growing cells. To determine if Top1-myc was destabilized in the early stages of aging, when the protein level was declining, we performed a CHX chase comparison between young and moderately aged yeast cells (∼6 generations). Top1-myc was again depleted in the aged cells, but there was still no significant turnover after 90 min in CHX (Fig. 2*E*). Based on these results, the depletion of Top1 during early stages of replicative aging is most likely due to reduced translation.

### Rescue of age-dependent Top1 depletion shortens RLS

**Figure 3:**
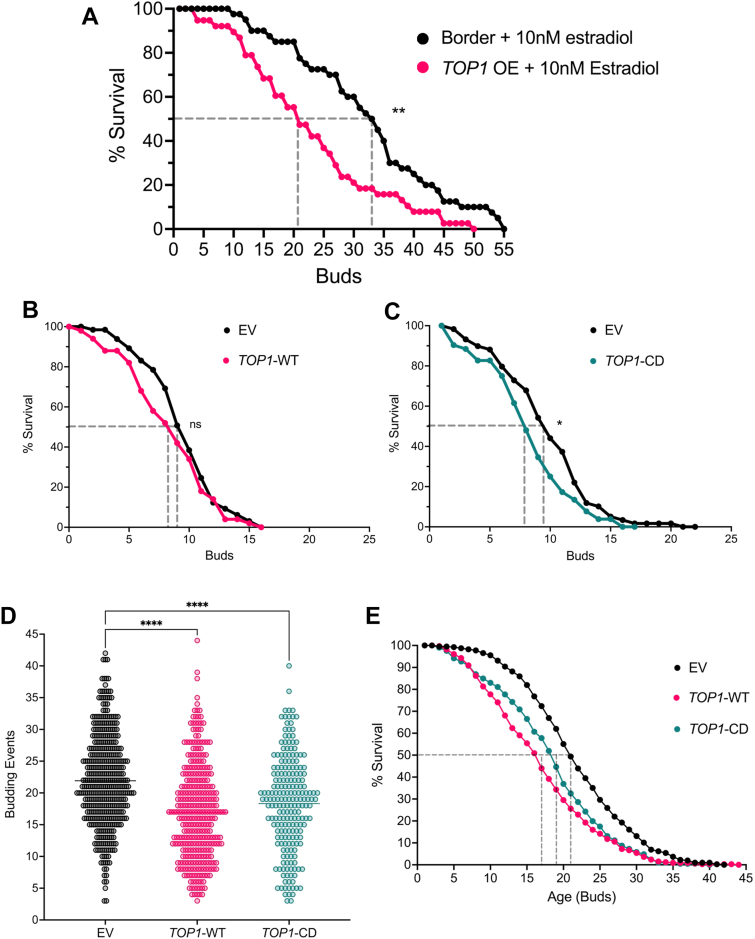
**Top1 overexpression to prevent age-associated depletion does not extend RLS**. *A*, RLS measured by manual microdissection for a WT border strain (LP55, n = 41 cells) and a Top1 YETI overexpression strain (LP56, n = 38 cells) on SC + 10 nM β-Estradiol agar plates. Mean RLS = 32.44 (LP55) and 22.79 (LP56), Log-rank test *p* = 0.0012. *B*, RLS manually counted from microfluidics chips with strains expressing either a low copy empty vector (LP181, n = 65 cells) or low copy vector expressing WT Top1 from its native promoter (LP183, n = 50 cells). Mean RLS = 9.55 (LP181) and 8.46 (LP183), *p* = 0.2519. *C*, RLS manually counted from microfluidics chips with strains expressing the empty vector (LP181, n = 59 cells) or catalytically dead (Top1-CD) mutant (LP185, n = 52 cells). Mean RLS = 9.85 (LP181) and 8.35 (LP185), *p* = 0.0354. *D*, automated counting of budding events for mother cells with an extra integrated copy of *SIR2* and harboring either a low copy pRS416 empty vector (LP257, n = 405 cells), a pRS416-WT-Top1 vector (LP263, n = 332 cells), or pRS416-Top1-CD vector (LP269, n = 206 cells). Mean RLS = 21.9 (EV), 16.9 (Top1-WT), and 18.3 (Top1-CD), Kruskal–Wallis test with Dunn’s test for pairwise comparisons with BH corrections for multiple hypotheses testing *p* < 0.0001. *E*, survival curve depiction of the automated budding counts for the same strains. Kruskal–Wallis rank-sum test *p* < 0.0001. BH, Benjamini–Hochberg; RLS, replicative lifespan; Top, topoisomerase; YETI, Yeast Estradiol strain with Titratable Expression.

Based on previous work showing that overexpression of either Mcd1 (a subunit of the cohesin complex) or Sir2 (a subunit of the RENT and SIR complexes) was sufficient to extend RLS (20, 30), we sought to test whether rescuing the age-dependent depletion of Top1 would also extend RLS. Top1 is important for recruiting Sir2 to the rDNA locus and deletion of Top1 causes silencing defects at the rDNA (15, 39), so we predicted that rescuing Top1 depletion would enhance silencing at the rDNA and extend RLS. Using a haploid strain from the Yeast Estradiol strain with Titratable Expression (YETI) collection that expresses Top1 tagged with 13xMyc (41), we observed ∼2-fold increased protein expression when induced with 2.5 or 5 nM β-estradiol as compared to the endogenous Top1-13xMyc level in a control (border) strain (Fig. S4). These strains were compared for RLS with a manual dissection assay on yeast peptone dextrose (YPD) agar containing 10 nM β-estradiol, and surprisingly, RLS of the *TOP1* overexpression strain was significantly lower than the border control strain (Fig. 3*A*). We hypothesized that the elevated Top1 expression could be detrimental due to DNA damage, which was described earlier for a highly expressed copper-inducible *TOP1* system (42). We therefore shifted to expressing *TOP1* expression from its own promoter on a low copy CEN/ARS *URA3* vector. Cells were grown in SC-Ura liquid media and RLS was tracked using an automated microfluidics imaging system, followed by manual counting of the cell divisions. This analysis still revealed a trend toward reduced RLS for WT-*TOP1* vector that was not statistically significant when compared to the empty vector (Fig. 3*B*). We therefore predicted that expressing a catalytically dead Top1-Y727F mutant (Top1-CD), which binds to DNA without forming cleavage-complexes, would help recruit Sir2 to the rDNA without inducing genome-wide damage, thus extending RLS (39). However, the same CEN/ARS plasmid expressing the Top1-CD mutant significantly decreased RLS compared to the empty vector control (Fig. 3*C*). We next considered the possibility that rescuing Top1 expression in aging cells did not extend RLS because Sir2 was also strongly depleted during aging, thus preventing the ability of Top1 WT or catalytically dead proteins to enhance rDNA silencing and RLS. To address this idea, we enhanced the Sir2 pool by integrating an extra copy of the *SIR2* gene at the *leu2Δ1* locus and then ran the microfluidics experiment again with the Top1-WT and Top1-CD *CEN/ARS* vectors. To our continued surprise, there was a significant decrease in RLS for both *TOP1* overexpression strains compared to the empty vector control (Fig. 3, *D* and *E*), as quantified by a custom-built automated image analysis pipeline (MicroBrew). The longer overall RLS observed for the survival curves in panel E compared to panels B and C is due to the increased *SIR2* expression, which strongly extends lifespan (20). Top1 overexpression therefore partially counteracts the RLS extension induced by increased *SIR2* expression.

### Overexpression of a catalytically dead Top1 mutant decreases silencing of an rDNA-flanking reporter gene

**Figure 4:**
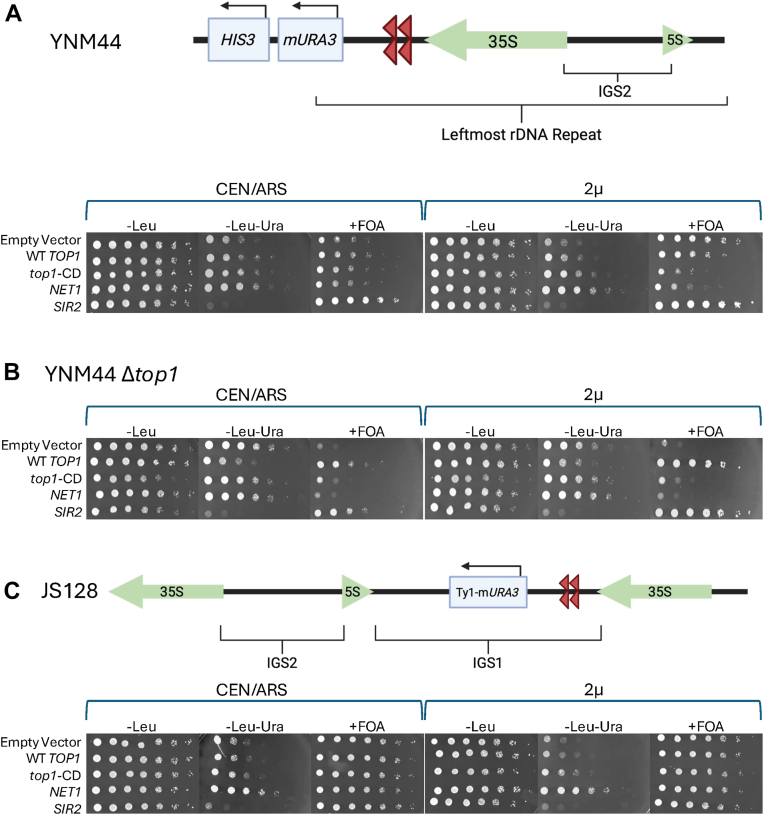
**Top1 overexpression negatively affects rDNA silencing**. Spot test growth assays on SC-Leu, SC-Leu-Ura, and SC-Leu + FOA plates to assess silencing of a *mURA3* reporter gene when *TOP1*, *top1-CD*, *NET1*, or *SIR2* are overexpressed from a low copy (CEN/ARS) or a high copy (2μ) *LEU2* vector. The plasmids were transformed into the following strains: *A*, YNM44, the *mURA3* reporter is directly flanking the rDNA leftmost repeat), (*B*) YNM44 *top1Δ*, (*C*) JS128, the Ty1-*mURA3* reporter was integrated at the IGS1 region of an rDNA repeat located within the tandem array). 5-FOA, 5-fluoroorotic acid; SC, synthetic complete; Top, topoisomerase.

Due to the negative effects of Top1 overexpression (both WT and catalytically dead) on RLS, we asked whether this overexpression affected rDNA silencing, as rDNA silencing is closely linked to RLS (18). To test the effects of Top1 overexpression on rDNA silencing, we performed silencing assays on strains carrying a modified *URA3* reporter gene (*mURA3*) integrated into unique chrXII sequence adjacent to the leftmost rDNA repeat (strain YNM44) (Fig. 4*A*) (43). Repressive chromatin spreads from the Fob1-binding sites (TER1 and TER2) into the adjacent *mURA3* reporter. In this assay, strains with weakened silencing of the *mURA3* reporter grow faster on SC media lacking uracil (SC-Leu-Ura) but slower on media that contains 5-fluoroorotic acid (SC-Leu+FOA). We transformed this reporter strain with low copy (CEN/ARS) and high copy (2μ) *LEU2* plasmids expressing either WT Top1 or catalytically dead Top1 from the native promoter and confirmed *TOP1* mRNA overexpression by qRT-PCR (Fig. S5). When YNM44 was transformed with either the CEN/ARS or the 2μ plasmid expressing WT *TOP1*, there was a slight weakening of silencing that became more severe with a 2μ Top1-CD catalytic mutant plasmid. This was indicated by increased growth on SC-Leu-Ura plates and weakened growth on SC-Leu+FOA plates (Fig. 4*A*). CEN/ARS and 2μ vectors expressing *NET1* or *SIR2* acted as negative and positive controls, as it has been published by our lab that *NET1* overexpression decreases rDNA silencing, while *SIR2* overexpression enhances rDNA silencing of the *mURA3* reporter (19). To confirm the changes in silencing observed as colony growth on plates, we also quantified growth rates for the 2μ set of reporter strains in liquid SC-Leu, SC-Leu-Ura, and SC-Leu+FOA cultures (Fig. S6). The relative growth rates observed in liquid culture assays mirrored those of the more traditional colony growth assay on plates. The silencing defect caused by WT *TOP1* overexpression was indeed milder than the catalytically dead mutant.

As previously discussed, strains with a *TOP1* deletion exhibit an rDNA silencing defect similar to that of *SIR2* deletion strains (15). To determine if expression of Top1-WT or Top1-CD from the plasmids was sufficient to rescue the *top1Δ* silencing defect phenotype, we deleted *TOP1* from YNM44 and transformed with empty vector, Top1-WT or Top1-CD plasmids. The WT Top1 CEN/ARS and 2μ plasmids both restored silencing as compared to the empty vector, indicated by reduced growth on SC-Leu-Ura and improved growth on SC-Leu+FOA (Fig. 4*B*). Interestingly, the catalytically dead Top1-CD did not restore rDNA silencing to the *top1Δ* strain, indicating that the catalytic activity of Top1 is important for establishing silencing. Previous studies reported that the catalytic activity of Top1 was not required for Sir2 recruitment to the rDNA (39), raising the question of why the activity would be required in the silencing reporter strains. This question will be addressed in the discussion.

Because overexpression of Top1 had negative effects on silencing of an *mURA3* reporter gene flanking the rDNA, we next asked if *TOP1* overexpression would also weaken silencing with the reporter gene positioned at the IGS1 region within the rDNA array. Previous literature has shown that RNA Pol II binding and ncRNA transcription at IGS1 were elevated when silencing of this region was lost *via* deletion of *sir2* (14). We therefore hypothesized that the Top1 overexpression vectors should also weaken reporter gene silencing at the internal IGS1 region. We found that when either WT Top1 or Top1-CD was overexpressed, silencing of the *mURA3* reporter within IGS1 was mildly derepressed (Fig. 4*C*). Like the left flanking reporter, overexpression of the Top1 catalytic mutant had a stronger effect on IGS1 reporter silencing than the WT Top1.

### Top1 overexpression removes Sir2 from the rDNA

**Figure 5:**
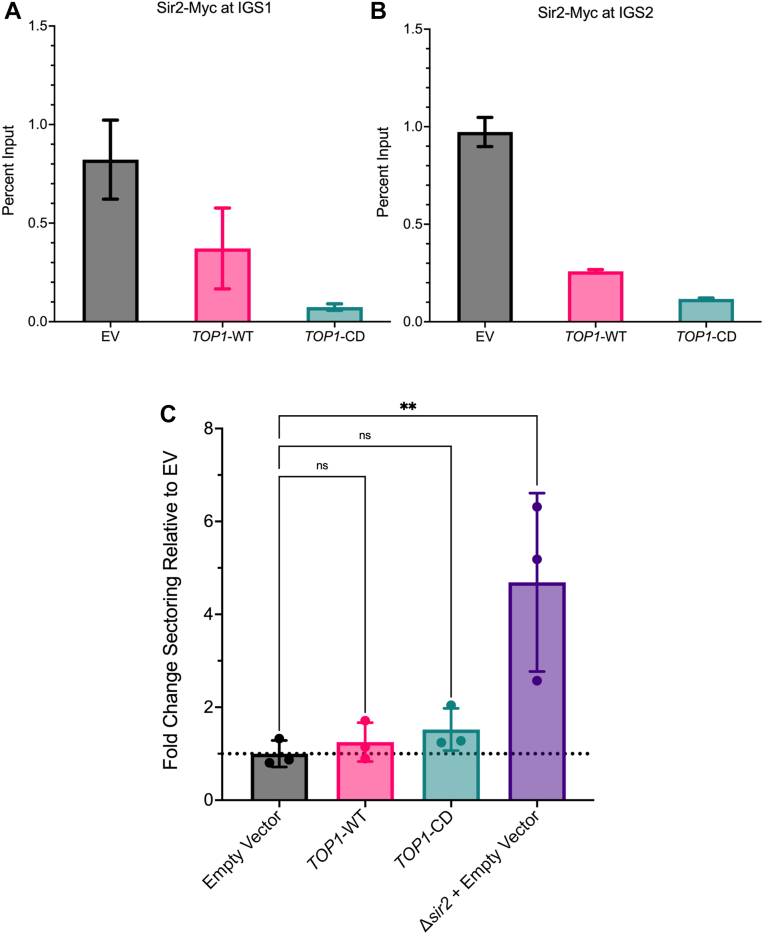
**Top1 overexpression reduces Sir2 binding at the rDNA and decreases rDNA stability**. *A*, quantitative ChIP-assay to assess enrichment of Sir2-Myc at the IGS1 region of rDNA, three technical replicates. *B*, quantitative ChIP assessing Sir2-Myc enrichment at IGS2. Strain MD209 harbors the empty vector (EV) pRS416, three technical replicates. MD208 harbors the Top1-WT vector, and MD210 harbors the Top1-CD vector. *C*, frequency of rDNA recombination indicated by loss of an integrated *ADE2* reporter. The frequency of half red/white sectored colonies from a strain (AW72) harboring the high copy 2μ empty vector pASC425 was normalized to 1.0. Additional overexpression strains were *TOP1*-WT (AW73) and *top1*-CD (AW74). A s*ir2Δ* deletion strain with the empty vector was used as a positive control for elevated sectoring (AW82). From *left* to *right p* = 0.9805, *p* = 0.8649, ∗∗*p* = 0.0056, one-way ANOVA with Dunnet’s correction for multiple comparisons. Three biological replicates were performed for each strain. ChIP, chromatin immunoprecipitation; Top, topoisomerase.

We next asked if the overexpression of Top1 or Top1-CD influenced binding of Sir2 at the intergenic spacer regions of the rDNA, thereby providing a mechanism for the weakened silencing and shortened RLS. Using chromatin immunoprecipitation (ChIP) assays, we found that overexpressing the WT Top1 or Top1-CD mutant from a low copy CEN/ARS vector was sufficient to significantly decrease the enrichment of Myc-tagged Sir2 at both IGS1 and IGS2, with the catalytically dead mutant having a greater effect (Fig. 5, *A* and *B*). This was consistent with the stronger negative effect of overexpressing the mutant on rDNA silencing.

The notable reduction of rDNA silencing, along with the disruption of Sir2 recruitment to the rDNA when Top1 was overexpressed, led us to ask if there were also negative effects on rDNA stability. To test this, we performed colony sectoring assays with a strain harboring an *ADE2* marker positioned within the rDNA array (20). Loss of this marker from the rDNA due to recombination repair events causes the cells to appear red when plated on media limited for adenine. Colonies that are half red and half white indicate that the *ADE2* marker was lost upon the first cell division after plating. The high copy WT Top1 and catalytically dead Top1 vectors were transformed into the *ADE2* reporter strain and then tested for marker loss, as compared to the empty vector. While not scored as significant, the *top1* mutant trended toward modestly elevated marker loss, consistent with the weaker rDNA silencing and displaced Sir2 phenotypes. As a positive control to ensure we can detect a significant increase in sectoring, we used a s*ir2Δ* strain known to have high rDNA instability (30) (Fig. 5*C*).

**Figure 6:**
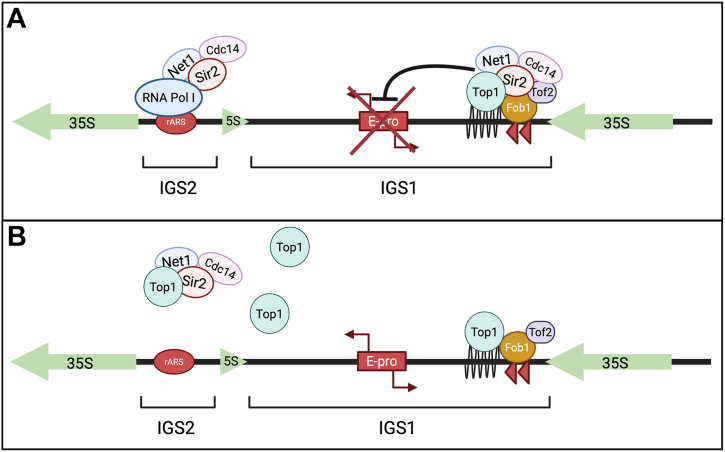
**Model for Top1 overexpression disrupting rDNA silencing and RLS**. *A*, under normal Top1 protein levels, RENT is recruited to IGS1 by interaction with Fob1 and to IGS2 by interaction with RNA Pol I at the rDNA promoter. Top1 catalytic activity enhances RENT enrichment and/or chromatin topology to establish silencing. *B*, when Top1 is overexpressed, the RENT complex is diluted away from the rDNA array and silencing of RNA Pol II transcription in the rDNA is lost, resulting in expression from E-pro. RENT, REgulator of Nucleolar silencing and Telophase exit; RLS, replicative lifespan; Top1, topoisomerase 1.

Given the role of Top1 catalytic activity in establishing rDNA silencing as well as its ability to dilute Sir2 away from both IGS1 and IGS2, we have proposed a model for the effects of Top1 overexpression at the rDNA locus (Fig. 6). In this model, under normal Top1 protein levels, Top1 is bound to Fob1 at the rDNA locus, where Top1 catalytic activity allows the RENT complex to bind and silence RNA Pol II transcription in the intergenic spacer (Fig. 6*A*). However, when *TOP1* is overexpressed, the RENT complex is diluted away from the rDNA array and silencing of RNA Pol II transcription in the rDNA is weakened (Fig. 6*B*), resulting in mild rDNA instability and shortened RLS.

## Discussion

### Age-dependent depletion of specific nuclear protein levels: a driver or a consequence of early aging?

While proteomic screens in young and old yeast cells have been reported previously in the literature, our proteomics screen was the first performed on both isolated nuclei and moderately aged cells (∼6–7 generations), providing novel insight into proteome changes occurring during the earliest stages of replicative aging. Consistent with previous whole cell aging proteome data showing a significant downregulation of ribosome assembly proteins in cells at ∼20 to 25 generations (36), we found a significant portion of the nuclear depleted proteins in early aging were involved in ribosome biogenesis, especially rRNA processing. Interestingly, our results with isolated nuclei support a decrease in biogenesis initiating even earlier than previously reported, at ∼6 to 7 generations. A decline in ribosome biogenesis will have critical effects on maintaining proteostasis, one of the hallmarks of aging (1). In addition to disrupting proteostasis, the early decline in ribosome biogenesis factors such as RNA Pol I subunits and rRNA processing factors could be directly contributing to the age-associated rDNA instability, since transcription of the rDNA by RNA Pol I is critical for establishing Sir2-dependent silencing and maintaining stability of the rDNA tandem array (19, 43).

Despite the decreases in ribosome biogenesis proteins exhibited in early aging cells, we still detect enrichment of numerous proteins in the aged cells, such as Hsp104, which was validated by Western blot analysis. Among the proteins enriched in the aged nuclear preparations, we found a significant increase in proteins involved in metabolism, consistent with previous literature that yeast cells will upregulate transcription of genes involved in gluconeogenesis and the glyoxylate cycle (44). The enrichment of specific proteins in early aging suggests that the previously observed general decrease in translation does not apply to all classes of proteins (26). Alternatively, the upregulated proteins could be accumulating due to increased transcription or protein stability. The mechanism of such selective protein enrichment therefore remains an unanswered question in the field.

Notably, we found that a large set of chromatin stabilizing factors are depleted in the early stages of aging. It is known that specific chromatin stability factors (Mcd1, Sir2, and Sir4) are depleted during aging and that this limited pool of proteins is redistributed from the rDNA to other regions of the genome (30). Through this screen we identified several chromatin stability proteins, in addition to Top1 discussed in this paper, that were significantly depleted with age such as Top2 and Rrm3 helicase, both of which warrant further study. Rrm3 in particular, plays a specific role in unwinding supercoiling at the rDNA replication fork block, where a replication fork and RNA Pol I transcription meet, and is critical for replication fork progression at the rDNA (45, 46). Despite these insights into how the nuclear proteome changes in early aging, it remains to be determined if these changes are causal for aging or simply a byproduct of the aging process.

### Mechanisms of Top1 depletion in early replicative aging

The mechanisms behind the observed age-dependent protein depletion remain unknown, and the specificity of which proteins become depleted raises intriguing questions for further exploration. Focusing on Top1 depletion specifically, we ruled out decreases in transcription or increased age-induced protein turnover as possible depletion mechanisms, though whether this is true for other depleted proteins needs more investigation. First, we concluded that during the early stages of replicative aging, Top1 mRNA transcript levels are not significantly changed, so transcriptional regulation of Top1 is unlikely to be the cause of Top1 protein depletion with aging. At the RNA-seq level, a recent study found that much of the altered individual protein abundance in old cells correlated with the change in mRNA abundance (47). However, Top1 provides a good example of an exception to this observation showing no change in mRNA abundance with age (Fig. 2*B*), despite the noted strong protein depletion. In budding yeast, many proteins are highly stable unless specifically involved in cell cycle regulation (48). Fitting with this data, Top1 is a highly stable protein, even in aged cells. Consistent with the hypothesis that aging-mediated depletion of Top1 is due to a decrease in translation, a previous ribosome-sequencing (Ribo-seq) study identified a decrease in Top1 translational efficiency during replicative aging (26). However, an independent Ribo-seq study indicated no age-associated change in Top1 (49). The discrepancy between the two ribo-seq findings could be due to differences in the average age of the cells or methods of mother cell isolation, so the exact mechanism of Top1 translational changes during aging warrant further study. Changes in nuclear transport during replicative aging could also affect Top1 levels in the cell. Supporting this idea, we observed significant depletion of Srm1, a GTP exchange factor necessary for proper mRNA and protein transport (50). Furthermore, stoichiometry of the nuclear pore is also known to be disrupted during replicative aging (51).

Lastly, recent studies have reported that increased cell size, a key phenotype of replicative aging yeast mother cells, could also impact proteostasis (52). Like our analysis of the nuclear proteome, recent whole cell stable isotope labeling by amino acids in cell culture proteomic analysis comparing young and old mother cells revealed significant reduction of histones and other chromatin associated factors in the old cells (47). Interestingly, such proteins were also reduced in *cln3Δ* cells (47), which have a large cell size due to slowed G1/S transition (53). This suggests that at least some of the age-associated protein abundance changes that we observe in nuclei could be related to the enlarged cell size. Interestingly, large *cln3Δ* cells accumulate ERCs as they are passaged for multiple generations (54), so the consequences on cell size and rDNA stability appear to overlap. Given that the cell size scaling of protein abundance is also conserved in mammalian cells (47), distinguishing chromatin regulatory processes impacted by cell size or ploidy changes from those specifically impacted by aging will be critical moving forward.

### Less is more: rescuing age-dependent protein depletion does not always extend RLS

Top1 plays a key role in the maintenance of rDNA stability. Top1 is recruited by Fob1 at IGS1 and enhances recruitment of Sir2 to facilitate rDNA silencing (38, 39). Furthermore, *TOP1* deletion has been shown to produce an rDNA instability phenotype (15, 55, 56, 57, 58, 59, 60). We were therefore surprised that rescuing the age-dependent depletion of Top1 did not extend RLS. This decrease in lifespan with overexpression could be due to an increase in DNA double-strand breaks from the formation of Top1 cleavage complexes where Top1 cuts the DNA but does not religate (42, 58). We therefore predicted that the structural role of Top1 in recruiting the RENT complex to the rDNA would be necessary for RLS extension but that too much of the catalytic activity of Top1 would be harmful. Surprisingly, the catalytically dead mutant decreased RLS even more than the WT or normal overexpression. Questions therefore remain about Top1 depletion in replicative aging. For example, does this depletion actually benefit the cells as they age because of a requirement to match the levels of key interacting multisubunit complexes that maintain rDNA stability, including RNA Pol I and the RENT complex (19, 27)? If the stoichiometry between Top1 and these complexes is drastically altered during aging, the rDNA could become more unstable and shorten RLS.

### Top1 plays a role in the establishment of silent chromatin at the rDNA locus through changes in chromatin architecture

To investigate the negative effects of Top1 on RLS, we looked at the impact of Top1 overexpression on an *mURA3* silencing reporter flanking the leftmost repeat of the rDNA, which is positioned adjacent to Fob1-binding sites (TER1/TER2) where the RENT complex is recruited (43). The benefit of using this reporter strain is that the *mURA3* reporter is integrated into unique chrXII sequence and not actual rDNA repeat sequence, meaning it is not subjected to the high level of recombinational instability that plagues silencing reporter genes integrated within the rDNA tandem array. *TOP1* overexpression, particularly high copy vectors, weakened silencing at this location. To confirm this effect, we also tested silencing of the *mURA3* reporter integrated at IGS1 within the tandem array, which likely has a different 3D chromatin environment. The weakened silencing effects were the same as observed for the rDNA flanking reporter, indicating that Top1 is likely functioning similarly at both positions.

Consistent with the loss of silencing, Top1 overexpression decreased Sir2 enrichment at the rDNA intergenic spacers (Fig. 5, *A* and *B*). This was consistent with the loss of silencing, but surprising given the scaffolding role for Top1 at the rDNA, which is still present even in the catalytic mutant (39). We therefore hypothesize that Top1 overexpression disrupts stoichiometry of the RENT complex by titrating Sir2 away from the complex (Fig. 6). Similarly, in strains where *NET1* has been overexpressed, rDNA silencing decreases, which is likely also from the disruption of RENT complex stoichiometry (19). WT Top1 overexpression from a high copy vector only caused a small decrease in rDNA silencing, while the catalytic mutant effect was much stronger. This could be explained by the larger effect the catalytic mutant Top1 has on blocking Sir2 from the rDNA that we observed in the ChIP experiments. Another possibility is that overexpression of excess WT Top1 is toxic to the cell, so cells are more likely to downregulate the endogenous *TOP1* promoter driving expression from the plasmid. When expressing a catalytic mutant, however, they do not have an increase in DNA damage that would trigger such a transcriptional feedback response, leaving more overall Top1 protein to compete Sir2 from the rDNA.

Lastly, we found that overexpression of Top1 from a high copy vector was sufficient to rescue the rDNA silencing defects of an endogenous *TOP1* gene deletion, but the catalytic mutant did not rescue the defects. The same was observed with a *CEN/ARS* set of *TOP1* and *top1-CD* plasmids. This is intriguing, as it indicates that the catalytic activity of Top1 is required for the establishment of rDNA silencing, a novel idea that remains underexplored. Interestingly, RNA Pol I transcription is required for the spreading of rDNA silencing repressive chromatin into the left flanking *mURA3* reporter, even though RNA Pol I transcription does not go past the rDNA genes (19). Additionally, Top1 is required for transcriptional elongation of RNA Pol I through rDNA genes (61). It is therefore possible that the positive supercoiling forced ahead of the transcription machinery, along with negative supercoiling accumulating behind RNA Pol I, produces a specialized DNA/chromatin topology downstream of the polymerase that favors RENT enrichment and contributes to the repression of RNA Pol II transcription (62). This hypothesis fits nicely with our data indicating that Top1 catalytic activity is necessary for the establishment of silencing at the left flanking *mURA3* reporter and suggests that the topology of the DNA/chromatin, not just the Sir2 recruitment by Top1, may be important to form the repressive chromatin domain. Supporting this hypothesis, at the silent mating-type loci on chromosome III, Sir2-dependent establishment of silent chromatin is more negatively supercoiled than nonsilenced chromatin (63). It makes sense then, that Top1 may be required to relieve the positive supercoiling ahead of the RNA Pol I machinery to establish repressive chromatin downstream that silences transcription by RNA Pol II. Additional work will be needed to elucidate this model for rDNA silencing establishment.

## Experimental procedures

### Yeast strains, plasmids, and media

Yeast strains were grown at 30 °C in YPD growth medium or synthetic complete (SC) dropout media for plasmid maintenance (64, 65). All strains are listed in Table S1, and all DNA plasmids and primers are listed in Tables S2 and S3 respectively. Proteins for western blotting were tagged with 13xMyc epitopes (EQKLISEEDL) at the C terminus of the endogenous locus (strains MD188, LP128, MD270, LP280) using PCR and *kanMX4* as a selectable marker for integration of the tag cassette with primers (Table S1). MD188, MD270, and LP280 were constructed using the method outlined in Longtine *et al*., 1998 (66). For LP128 and LP124, a *TOP1*-13xMyc cassette was PCR amplified from YRH919 genomic DNA, which already harbored the insertion, before transformation into BY4741 and LP56, respectively. Strain LP56 is from the YETI overexpression collection and has an estradiol inducible promoter upstream of the endogenous *TOP1* gene (41). Titratable overexpression of Top1 protein from strain LP124 was confirmed with Western Blots. Tagged strains were verified with both colony PCR and Western blots. The high copy 2μ Top1-LEU2 (pAW6) and 2μ Top1-CD-LEU2 (pAW8) vectors were constructed by PCR amplifying the WT *TOP1* or catalytic mutant *top1*-CD genes with their native promoters and 3′ termination sequences from plasmids pNK66 and pNK67, respectively (67). PCR products from primers JS4054 and JS4055 were digested with *Asc*I restriction endonuclease and ligated into plasmid pAsc425 (19). Low copy versions of these plasmids were generated (CEN/ARS *TOP1-LEU2* (pAW2) and CEN/ARS *top1-*CD*-LEU2* (pAW4)) by ligating the same PCR products into pAsc415. Plasmids were verified with restriction digests and Sanger sequencing. The *NET1 CEN/ARS LEU2* plasmid pSB794, *SIR2 CEN/ARS LEU2* plasmid pSB764, *NET1 2μ LEU2* plasmid pSB790, and *SIR2 2μ LEU2* plasmid pSB766 were previously described and constructed similarly with PCR and *AscI* ligation into pAsc415 or pAsc425 (19). Strains transformed with plasmids were maintained in selective media to prevent plasmid loss.

### Nuclei isolation for proteomic screen

Approximately 4 × 10^8^ young or aged cells isolated from the MAD (see description below) and nuclei purified using a Yeast Nuclei Isolation Kit (ab206997) from Abcam following the kit procedure with minor modifications. Cells were washed two times with distilled water with centrifugation at 3000 × *g* at room temperature, then resuspended in 1 ml of buffer A containing 10 mM DTT and incubated at 30 °C for 10 min with gentle shaking. The samples were centrifuged at 1500 × g for 5 min at room temperature and the supernatant discarded. The pellets were resuspended in 1 ml of buffer B, followed by addition of 10 μl of Lysis Enzyme Cocktail and incubation of 15 min at 30 °C for young cells and 18 min for old cells to generate spheroplasts. The samples were then centrifuged at 1500 × *g* for 5 min and the pellets were kept on ice after discarding the supernatants. The spheroplast pellets were resuspended in 1 ml of buffer C containing protease inhibitor cocktail from the kit and then homogenized in a Dounce homogenizer. The suspension was mixed by shaking for 30 min at room temperature then centrifuged at 1500 × *g* for 5 min at 4 °C to remove cell debris. Supernatants were collected and then centrifuged at 20000 × *g* for 30 min at 4° to collect the nuclei, which were resuspended in buffer C. Purified nuclei were quantified for protein concentration and visualized by 4′,6-diamidino-2-phenylindole staining under a fluorescence microscope. H3-dependent HAT activity is nuclear specific in yeast, so enzymatic HAT activity from the nuclei was also validated using H3 substrate following the manufacturer's protocol with provided nuclear extract (4 mg/ml) as a positive control.

### TMT mass spectrometry

TMT labeling of nuclear proteins was performed in the biomolecular analysis facility core in the UVA School of Medicine following a standard protocol from the Gygi lab (68), with changes noted here. Protein extracts in 50 mM HEPES pH 8.2, 8 M urea, 50 mM NaCl, 1x protease inhibitor cocktail (Complete Mini, Roche) were generated from bead beating the nuclei then clarifying by centrifugation at 13,000 rpm for 15 min. Protein was quantified with a Qubit 3.0 fluorometer (Thermo Fisher Scientific). Approximately 100 μg of protein from each sample was reduced with 10 mM DTT at room temperature for 45 min, followed by alkylation with 10 mM iodoacetamide for 30 min in the dark at room temperature. Methanol/chloroform precipitation was performed immediately following alkylation, and the protein pellets were washed 3x with ice-cold methanol and rapidly dried by vacuum centrifugation. The sample pellets were resuspended in 20 μl of 50 mM ammonium bicarbonate solution pH 7.8 by repeated pipetting on ice and digested with an approximate 25:1 sequencing grade trypsin at room temperature for 16 h. The reaction was stopped by the addition of 2.5 mM TFA to a pH < 2 and lyophilized with vacuum centrifugation. The peptides were resuspended in 0.1% TFA and verified at a pH of 3 before peptide desalting with spin tips (Pierce C18 Spin Tips and Columns) following manufacturer protocols and again lyophilized. Individual samples were labeled with TMT reagents resuspended in anhydrous acetonitrile at a ratio of 1:4 peptide to TMT reagent. The labeled peptides were individually desalted to remove excess TMT reagent, dried with vacuum centrifugation and combined to a total volume of 40 μl in 0.1% TFA. Mass spectrometry was run on an LC-MS (Thermo Electron Q Exactive HF-Xmass spectrometer) system with an Easy Spray ion source connected to a Thermo 75 μm × 15 cm C18 Easy Spray column. 15 μl of the extract was injected and the peptides were eluted from the column by an acetonitrile/0.1 M formic acid gradient at a flow rate of 0.3 μl/min over 2.0 h. The nanospray ion source was operated at 1.9 kV. The digest was analyzed using the rapid switching capability of the instrument acquiring a full scan mass spectrum (120 K resolution) to determine peptide molecular weights followed by production spectra (30 high-energy collision dissociation at 7.5 K resolution, 100 ms fill, 35 normalized collision energy, 1.4 Da isolation) to determine amino acid sequence in sequential scans. The data were analyzed by database searching using the Sequest search algorithm in Proteome Discoverer 2.2 against UniProt yeast (10/23/19). Identifications and TMT reporter quantification were performed in Scaffold. The samples produced identifications for ∼1000 proteins. The log fold change between young and old cells was calculated and adjusted *p* values were calculated. Analyzed results of proteomic data are available in Table S5.

### Isolation of aged yeast cells

A MAD was set up based on designs and protocols from the Dunham Lab and Calico Labs (32, 69). 300 ml cultures were grown shaking in YPD at 30 °C overnight until an A_600_ of ∼1.2 to 1.7 was reached (log phase). Cells were pelleted and counted by hemocytometer. For each mini-chemostat tube (each chemostat run has 12 tubes), 250 million log phase yeast cells were washed 3 times with 1x PBS/0.25% PEG-3350. Cells were resuspended in 500 μl of 1xPBS, and then 500 μl of PBS with 4 mg/ml reactive biotin (Thermo-Scientific EZ-Link Sulfo-NHS-LC-Biotin) was added. Cells were biotinylated for 30 min with slow rotation at room temperature and then washed twice with 1xPBS to remove unbound biotin. Cells were transferred to 50 ml of YPD media and incubated while shaking at 30 °C for 3 h to recover. Cells were then pelleted and washed with 1xPBS, then resuspended in 600 μl of 1xPBS. Fifty microliters of magnetic-streptavidin beads (Invitrogen Dynabeads MyOne Streptavidin C) suspended in PBS were added to each 600 μl of biotinylated cells and incubated with slow rotation at room temperature for 30 min. After bead binding, tubes were placed on a magnetic rack, and cells that did not have any beads bound (unbiotinylated daughter cells) were removed. These unbiotinylated daughter cells were kept as the “young control.” The remaining bead-bound cells were loaded into the chemostat tubes and bound to the sides by strong neodymium magnets. Mother cells were incubated in the chemostat device for the indicated amount of time with fresh SC media continuously added with a Watson Marlow 205S peristaltic pump and aerated by aquarium air pumps. Air flow also keeps the daughter cells from settling at the bottom, so they are washed away into waste containers. After aging the mother cells, the content of each chemostat tube was harvested, and magnetic racks were used to collect the mother cells and wash away remaining daughter cells. The average age of the mother cells was determined using calcofluor white staining (incubated in 1 mg/ml calcofluor for 15 min, washed and resuspended in 1xPBS), and imaged at 60x objective with an EVOS M7000 microscope from Invitrogen (Thermo Fisher Scientific).

### Real-time PCR (RT-PCR)

Total RNA from ∼1 × 10^8^ cells was isolated using the hot acid phenol method and resuspended in diethyl pyrocarbonate-treated water (70). One microgram of RNA was used for complementary DNA (cDNA) synthesis with the Verso cDNA Synthesis Kit (Thermo Fisher Scientific) and cDNAwas diluted 3-fold in nuclease-free water. qRT-PCR to determine *TOP1* transcript levels was performed on an Applied Biosystems StepOnePlus Real-Time PCR system with primers JS3398 and JS3399. Log fold change was calculated using the ΔΔC_t_ method, where *TOP1* signal was normalized to *UBC6* signal (primers JS3976 and JS3977), and the normalized *TOP1* from old cells was compared to the average of the young control. qRT-PCR was performed on 3 biological replicates.

### Western blots

Total protein extracts from ∼5 × 10^8^ cells were isolated from thawed cell pellets using a protocol adapted from Dunn and Wobbe (71). Pellets were resuspended in 400 μl of lysis buffer (50 mM Hepes pH 7.6, 10% Glycerol, 10 mM EDTA, 0.5 M NaCl, 1% Triton X-100, 5 mM DTT, 1X protease inhibitor cocktail, 1 mM PMSF) and lysed with an equal volume of acid washed glass beads by vortexing (MPI Fastprep-24 classic bead beating lysis system) for 4 × 45 s with 1 min of rest in between (only 3 × 45 s for old cells). Lysates were cleared by centrifugation at 14,000 rpm for 10 min in an Eppendorf microcentrifuge at 4 °C, and supernatants were transferred to fresh microfuge tubes. Protein concentration was measured by Bradford Assay with a UV-visible spectrophotometer (BioMate3, Thermo Fisher Scientific). One volume of 5x Laemmli buffer was added to 4 volumes of cleared protein lysate. A volume of lysate containing 20 μg of protein was loaded onto 10% SDS-PAGE gels and run for ∼90 min at 120 V. Protein was transferred to a polyvinylidene difluoride membrane (Millipore Sigma Immobilon-P) by wet transfer in 1x Tris-Glycine buffer at 30 V for 16 h at 4 °C in a Biorad mini-gel tank. Membranes were blocked in 5% nonfat dried milk/1x Tris-buffered saline with Tween-20 (TBST) for 1 h at room temperature. Primary antibodies such as c-Myc mAb (9E10), Vma2 mAb (13D11B2, RRID AB_2536202), GAPDH Loading Control mAb (GA1R, RRID AB_10977387), or alpha tubulin mAb (YL1/2, RRID AB_2210201) were diluted 1:5000 in the milk/1xTBST blocking solution and incubated/rocked with the immobilon membrane for 1 h at room temperature. Membranes were next washed 3 × 5 min in 1xTBST then incubated in secondary antibody (Anti-Mouse IgG (H + L), horse radish peroxidase conjugate, Promega) diluted 1:5000 in 5% skim milk/1xTBST for 45 min at room temperature. Membranes were washed again, and protein bands of interest were visualized by chemiluminescence (Immobilon Western Chemiluminescent horse radish peroxidase Substrate) on a Bio-Rad Chemidoc MP system using the automatic exposure settings. Molecular weights were estimated using a protein ladder (Precision Plus Protein Dual Color Standards, Bio-Rad). Images were quantified in ImageJ (https://imagej.net/ij/), where boxes of equal size were used to measure band intensity and background. The background-subtracted 13xMyc bands were normalized to the respective loading control signal (also background corrected). The change in 13xMyc signal for each sample was normalized to the mean of the young cells. Western blots for age-dependent protein changes were performed with 3 biological replicates, and representative images are shown.

### CHX chase assays

CHX chase assays were performed according to a protocol from the Rubenstein lab (72). Time points for CHX chase assays were taken at 0, 30, 60, and 90 min or 0, 60, 120, and 240 min. For CHX chase assays with aged cells, samples were harvested directly from the chemostat and immediately subjected to CHX incubation. After CHX incubation at 250 μg/ml, cells were pelleted and frozen on dry ice. Frozen pellets (∼2.5 A_600_ units) were used for Western blotting *via* the protocol previously described. Quantification was performed as previously described with GAPDH or Tub1 loading controls for young cells and Vma2 as the loading control for aged cells. All CHX chase assays were quantified with 3 biological replicates, and representative Western blot images are shown.

### RLS assays

Traditional manual RLS plate assays were performed on agar plates based on a protocol from the Kaeberlein lab (73). Approximately 50 dividing cells from each strain were arranged in a grid on SC agar plates containing 10 nM β-estradiol (for overexpression with the YETI strains) using a Nikon Eclipse E400 microscope outfitted with a manual micromanipulator and fiber optic dissection needle. After being placed on the agar plates and incubated for ∼1 h, the mother cells are removed to the side of the plate and the new virgin daughter cells become the cohort of mother cells to be used for the experiment. Microdissections were performed every hour by pulling the smaller daughter cell from the mother cell and moving it to the side of the plate with the microdissection needle. In between generations, plates were incubated at 30 °C and then moved to 4 °C each night to stop the cell division until continuing in the morning. Mother cells that stopped budding within the first 2 generations were censored to avoid incorporating already dead cells. Once a mother cell ceased dividing for three generations, it was marked as dead. Plates were monitored until all mother cells had stopped dividing.

### Microfluidics RLS assays

Cells were grown to log phase in SC-Ura media to maintain the *TOP1* expression plasmid or empty control plasmids that harbored a *URA3* selectable marker. Cells were diluted to an A_600_ of 0.1 in filtered SC-Ura media and loaded into the Y-shaped traps of the commercial microfluidics chips that are embedded on standard size microscope slides (iBiochip Automated Dissection Kit). SC-Ura media was run through the microfluidics chip at a constant flow of 2 μl/min to provide fresh media to the cells and wash away the new daughter cells. The chips/slides were placed in an onstage incubator set to 30 °C. The Y-shaped nature of the traps in the microfluidics chip ensured that the cells trapped were virgin daughter cells (74). Images of each well were taken every 10 min with the 40x objective on an EVOS M7000 motorized stage microscope (Invitrogen, Thermo Fisher Scientific). Images were concatenated into time lapse videos using the Image-J import image sequence function and saved as AVI files. For manual counting of budding events from the movies, the AVI file names were blinded, and budding events were counted for ∼60 mother cells per condition. Budding events were only counted for mother cells that entered the trap within the first 12 h of the experiment to avoid counting buds for the daughter cells derived from old mothers. Traps where multiple cells had entered were also discarded to avoid incorrect counting of budding events. For both the plate assays and the manually counted microfluidics videos, Kaplan–Meier survival curves were generated using OASIS2 (75). Significant differences in lifespan were calculated using a log-rank test. For microfluidics experiments that were analyzed with automated counting, we used a custom-built program described below. Significant differences in lifespan were calculated in MicroBREW using a Kruskal–Wallis test to compare three or more independent groups without assuming normality and followed the test with Dunn’s *t* test for pairwise comparisons with Bonferoni corrections for multiple hypotheses testing.

To quantify RLS using a high-content imaging platform, we developed a custom image processing pipeline in MATLAB named MicroBREW. The analysis was performed in two main steps. In the first step, each image frame was processed to detect and record the positions of individual microfluidic traps. Briefly, edge detection (“*edge*,” default parameters) was applied to identify trap boundaries, and the resulting images were converted to binary format. Morphological operations, including dilation and hole filling (“*imfill*,” default settings), were used to refine object structures. Labeled binary images were then generated using “*bwlabel*” to assign unique identifiers to each detected object. A size threshold filter was applied to exclude large artifacts, such as those arising from chains of budded cells. For each identified trap, spatial coordinates (x, y) were extracted and used to track the trap location consistently across all frames in the time-lapse movie.

In the second step, four regions of interest (ROIs) were defined for each trap based on the spatial coordinates. These included one central circular region to track the entry and position of the mother cell, and three adjacent kite-shaped regions to capture signals associated with budding events. Within each of the four ROIs, background was corrected by subtracting the mode of the signal intensity distribution, assigning zeros if the values resulted in negative. The background-corrected pixel intensities were then aggregated and mapped to the corresponding trap ID and frame number for downstream analysis.

Signals generated in MATLAB were further processed in R to identify mother cell entry, cell death, and budding events for each trap. For each trap, the signal from the central ROI was first smoothed using a simple moving average and normalized to the pixel intensity in the first frame of the time series. Peaks in the normalized signal were detected using the “*findpeaks”* function with a stringent threshold to reduce false positives due to noise. The first identified peak marked the time of mother cell entry, and the corresponding frame ID was recorded. To detect budding events, the signals from the three kite-shaped ROIs were smoothed (*post entry*) using a moving average, and peaks were identified using “*findpeaks*,” with a minimum peak distance of six frames to reflect the expected time between budding events. Mother cell death was inferred from the central ROI by calculating the first-order lag difference; a sustained drop in the signal was used to indicate cell death. For each trap, the number of budding events detected between the mother cell entry and death was recorded per ROI. Additionally, the start and end points of each peak were captured to eliminate overlapping events, and peak heights were recorded to estimate the magnitude (size) of each budding event.

### Silencing assays

Cells were patched onto SC-Leu agar plates and incubated overnight at 30 °C for ∼24 h. Cells were scraped up from the agar surface and resuspended in 1 ml of sterile water in microfuge tubes. The A_600_ was measured with a UV-visible spectrophotometer (BioMate3, Thermo Fisher Scientific), and each sample was subsequently diluted and normalized to an A_600_ of 1. A series of 1:5 dilutions of cells in sterile water was made for a total of 7 dilutions. Five microliters of each dilution was then spotted in rows onto SC-Leu, SC-Leu-Ura, or SC-Leu + FOA agar plates to track the level of silencing of a modified *URA3* reporter gene (*mURA3*) that was integrated either adjacent to the rDNA locus or within the IGS1 intergenic spacer (16, 43). Plates were incubated for 72 h at 30 °C. Images to visualize silencing effects were taken with a Fluorochem-Q imager (Protein Simple).

For quantitative growth curve silencing assays, each strain was grown overnight in SC-Leu media and then normalized to A_600_ of 1.0. Next, 9 μl was added to 166 μl of either SC-Leu, SC-Leu-Ura, or SC-Leu + FOA media in 96-well plates. The plates were incubated in a BioTek LogPhase 600 Microbiology Reader (Agilent), which was set to read the OD_600_ every 10 min over 48 h for SC-Leu and SC-Leu + FOA, or 72 h for the SC-Leu-Ura samples. Plates were incubated at 30 °C while shaking at 800 RPM. The experiment was run with 3 biological replicates. Growth rate was extrapolated from the exponential growth phase of each curve. Fold changes were calculated relative to the rates of each strain in the SC-Leu control media and then normalized to the empty vector control strain in the matching media.

### ChIP assays

ChIP was performed based on a standard protocol used in the Smith lab (76). Briefly, log phase yeast cells were crosslinked with 1% formaldehyde for 20 min, pelleted, washed, and resuspended in lysis buffer (50 mM Hepes, 140 mM NaCl, 1% Triton X-100, 1 mM EDTA, 0.1% SDS, 0.1 mM PMSF, and 1X protease inhibitor cocktail; Sigma [Sigma Chemical]). The cell mixtures were then disrupted with glass beads, followed by sonication in a Diagenode Bioruptor for 15 min with 30 s on/30 s off cycles, followed by centrifugation at full speed in an Eppendorf microcentrifuge to collect the soluble supernatants. The protein content of the cell lysate was measured using Pierce Bradford Plus protein assay reagent (Thermo Fisher Scientific #1856209) and 1 mg of the chromatin lysate was used for each ChIP sample. For Myc-tagged samples, 5 μl of 9E10 anti-Myc antibody (EMD Millipore, catalog #05-419) was used for immunoprecipitation overnight at 4 °C, and 1/10th of the supernatant volume was kept as input. The next day, immunoprecipitated lysates were incubated with 25 μl protein G magnetic beads (Thermo Fisher Scientific #88848) for 2 h at 4 °C followed by washing. The DNA was then eluted in elution buffer and reverse-crosslinked overnight at 65 °C. DNA samples were purified using PureLink PCR spin columns (Invitrogen). ChIP DNA was quantified using real-time PCR (Step-One Plus Applied Biosystem) and normalized to the input DNA PCR signal. The percentage (input %) value for each sample is calculated using % of input = 100∗2^(adjusted input – Ct(sample))^. Three technical replicates of ChIP assays were performed with 1 biological replicate.

### Sectoring assays

Cells were grown to log phase overnight in SC-Leu liquid media. Overnight cultures were pelleted and then washed with sterile water. Cells were then counted by hemocytometer and diluted such that when 100 μl of the diluted cells were plated onto SC-Leu with a limiting concentration of 80 μM adenine, yielding ∼400 colonies/plate. Three biological replicates from different colonies were spread onto 50 plates for each strain. The agar plates were incubated at 30 °C for 72 h, then 4 °C for 24 h to fully develop the red coloring of the Ade^-^ cells. Half sectors (half white/half red colonies) and one-fourth sectors (one-fourth red/three-fourths white colonies) indicating a loss of *ADE2* after the first or second cell division were counted using an Olympus SZ-PT microscope. The total number of colonies per plate was automatically counted using OrganoSeg (77). Entirely red colonies were excluded from total colony counts as they had lost the *ADE2* marker before the first cell division. The percent sectoring frequency for each plate was calculated using the equation below. The fold change in sectoring frequency was calculated relative to the mean of the empty vector control. The sectoring assay was performed on 3 biological replicates in 3 independent experiments.

$$\%\;Sectoring\;Frequency=\frac{\#\;of\;sectored\;colonies\;}{\#\;of\;white\;colonies\;+\;\#\;of\;sectored\;colonies}*100$$

### Statistical analyses

Statistical analysis for the proteomic screen was performed using a one-way ANOVA with corrections for multiple comparisons to find significant differences (*p* < 0.05) in protein levels of moderately aged cells compared to old cells. Five biological replicates of aged cell populations were compared to 5 biological replicates of young cell populations. For RT-qPCR and Western blot comparisons of relative mRNA fold change or protein abundance, we performed *t*-tests (two groups) or one-way ANOVAs (>2 groups) with Dunnett’s test for multiple comparisons. We normalized protein or mRNA abundance to the average of the control group for each value and compared the treatment groups to the control group for each statistical analysis. For Western blots and qPCR, we used 3 biological replicates per treatment group. For the sectoring assay, we normalized the sectoring frequency of each sample to the mean sectoring frequency of the empty vector control group and compared experimental groups to the control group using one-way ANOVA with corrections Dunnett’s test for multiple comparisons. For comparison of RLS survival curves we performed log-rank tests. Comparison of mean RLS was performed by Kruskal–Wallis test with Dunn’s test for pairwise comparisons and with Benjamini–Hochberg corrections for multiple hypotheses testing. Graphical representations of *p* values are pictured as follows: (ns = *p* ≥ 0.05, ∗ = *p* < 0.05, ∗∗ = *p* < 0.01, ∗∗∗ = *p* < 0.001, and ∗∗∗∗ = *p* < 0.0001).

## Data availability

Data supporting the findings of this study are available within the article or Supplementary Information files. Raw proteomics data is available at ProteomeXchange *via* the PRIDE database, accession number PXD066806.

## Supporting information

This article contains supporting information.

## Conflict of interests

The authors declare that they have no conflicts of interest with the contents of this article.
