# Supplementary material for: Age-dependent topoisomerase I depletion alters recruitment of rDNA silencing complexes
Source: J Biol Chem. 2025 Dec 17;302(2):111062. doi: 10.1016/j.jbc.2025.111062 (PMC12834909; doi:10.1016/j.jbc.2025.111062)
Supplement: Supplementary Material 1 [file mmc1.pdf]

Supporting Information

Age-dependent topoisomerase I depletion alters recruitment of rDNA silencing complexes.

Lindsey N. Power, Natalia Zawrotna, Manikarna Dinda, Abigail E. Weir, Bishal P. Paudel, Oshil Ghimire, Karolina Kisiel, Christopher T. Letai, Kevin A. Janes, Jeffrey S. Smith.

Figures S1-S6

Tables S1-S4

Excel File:

Table S5. Complete proteomic dataset comparing protein levels of isolated nuclei from replicatively aged (~6-7 generations) and young (~0-2 generations) yeast cells using TMT-MS.

Figure S1. **(A)** Representative image of DAPI stained nuclei isolated from strain SY38. **(B)** Histone acetylase (HAT) activity assay performed on isolated nuclei from young and old cells, and a positive control using HeLa cell nuclear extract (4 mg/ml). ****$p<0.0001$ (one-way ANOVA with Dunnett's correction for multiple comparisons compared to positive control, $n=3$ technical replicates for control and $n=10$ technical replicates for young and old cells).

A

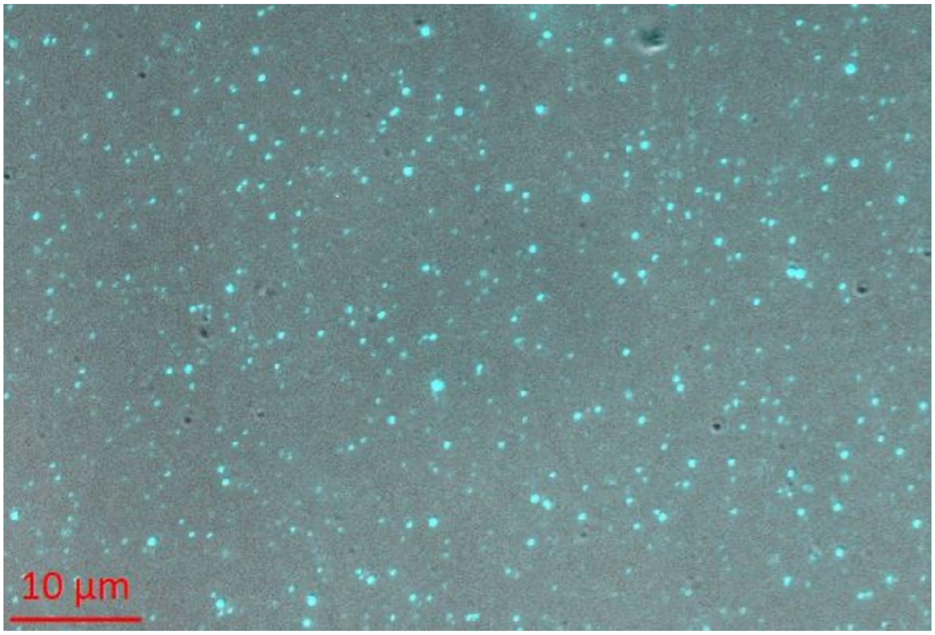
10 µm

B

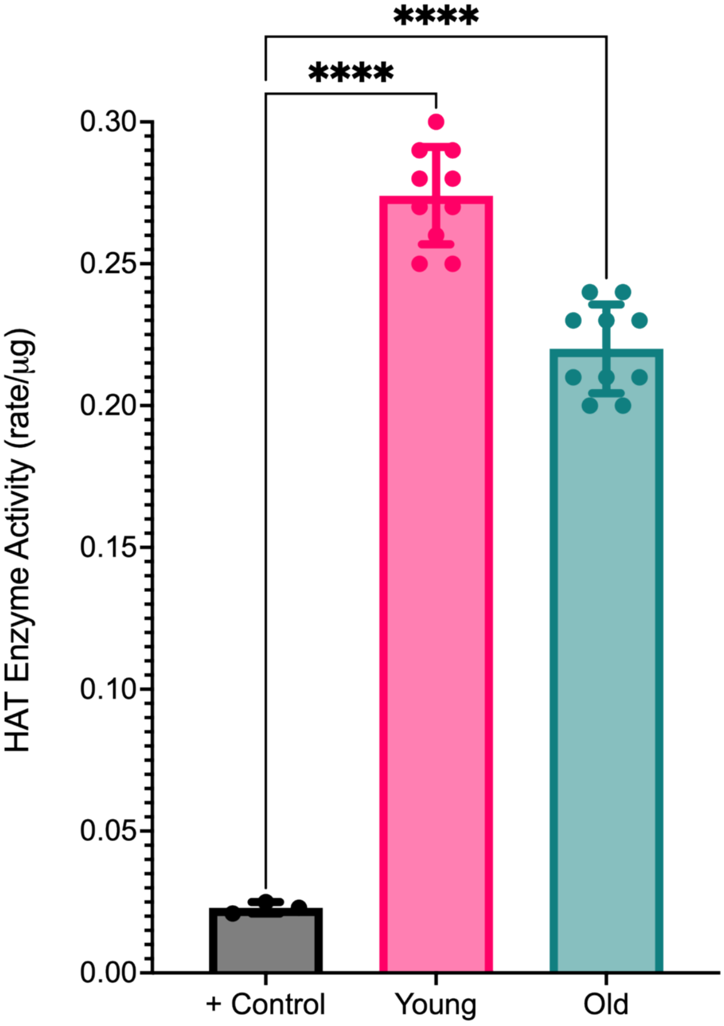
****
****
HAT Enzyme Activity (rate/µg)
0.30
0.25
0.20
0.15
0.10
0.05
0.00
+ Control
Young
Old

Figure S2. Quantitative RT-PCR of lncRNA expression from the rDNA IGS1. The fold change relative to WT (BY4741) is indicated for Top1-Myc tagged (LP128) or *sir2*Δ (SY533) strains. (One-way ANOVA with Dunnett's test for multiple comparisons, p= 0.9812, *p=0.0171, n=3 biological replicates).

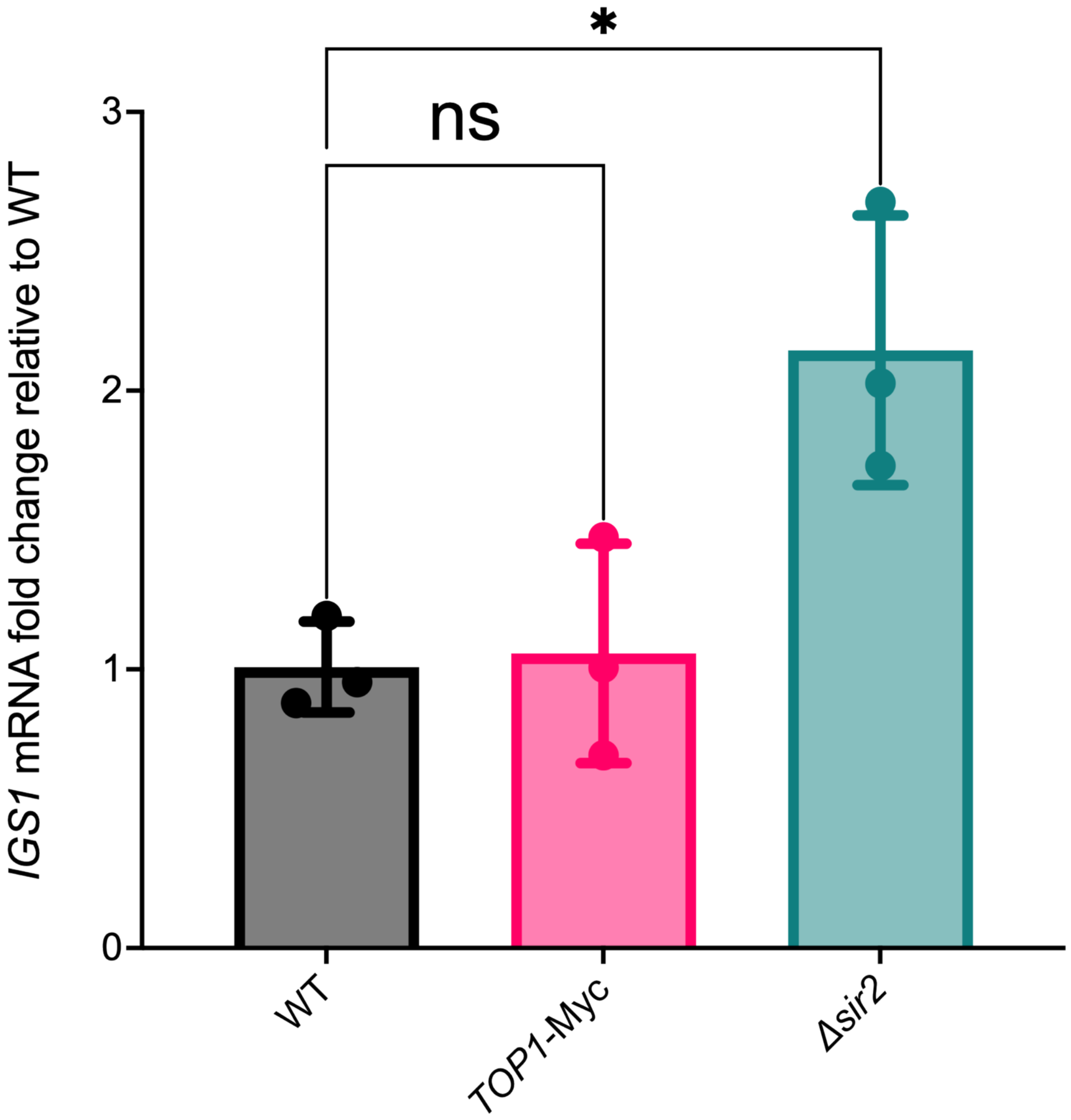

Figure S3. Cycloheximide chase experiment validation. **(A)** Growth curve measured by OD_{600} of wildtype yeast strain BY4741 grown in liquid YPD media with a final concentration of 0, 125, 250, 250, or 500 µg/mL cycloheximide. **(B)** Cdc13-13xMyc protein levels after 30, 60, 90, 120, or 240 minutes of growth after addition of 250 µg/mL cycloheximide at non-permissive temperature (30°C). GAPDH is used as the loading control.

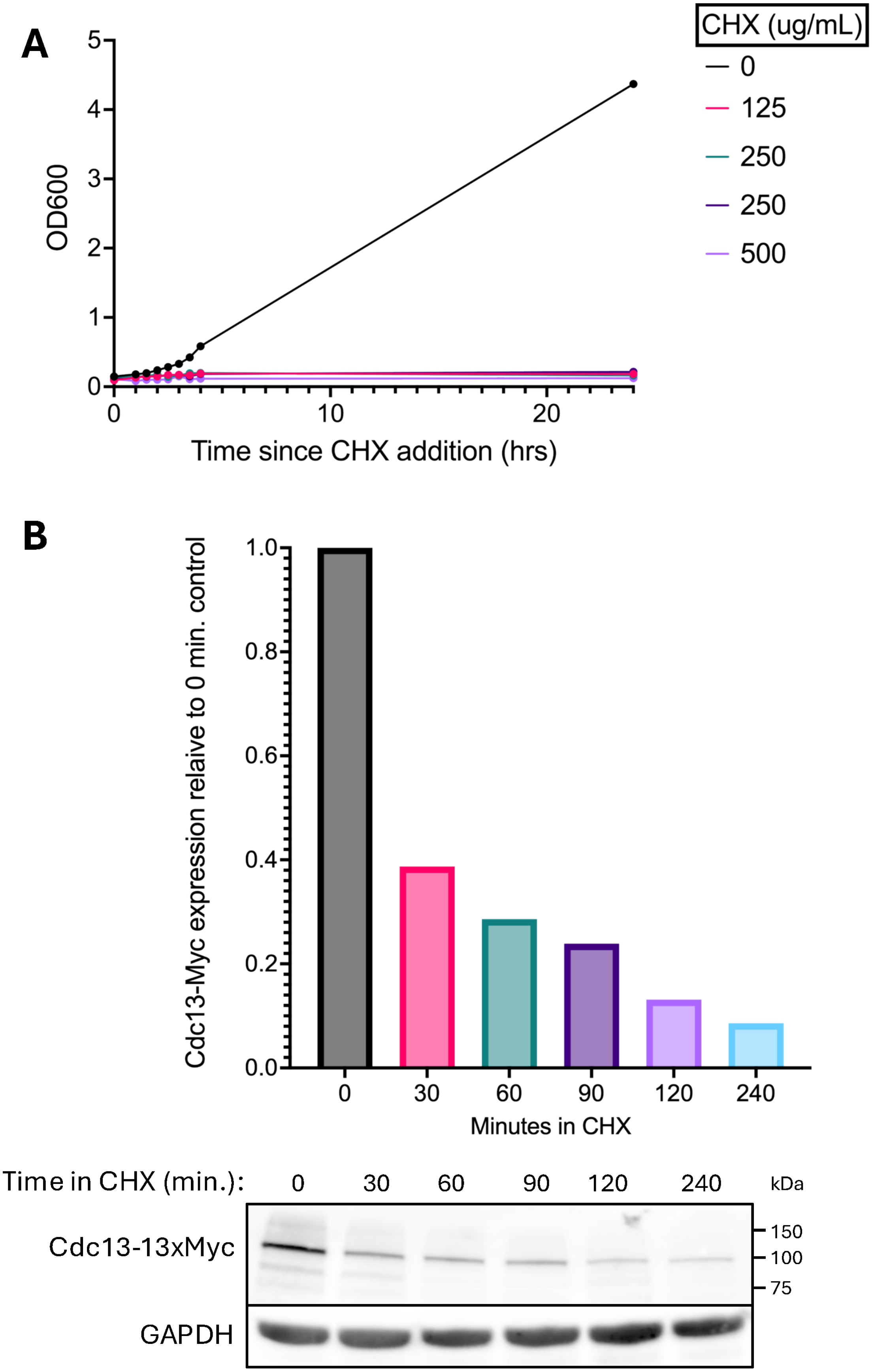
A
5
4
3
2
1
0
OD600
0
10
20
Time since CHX addition (hrs)
CHX (ug/mL)
0
125
250
250
500
B
1.0
0.8
0.6
0.4
0.2
0.0
Cdc13-Myc expression relaive to 0 min. control
0
30
60
90
120
240
Minutes in CHX
Time in CHX (min.):
0
30
60
90
120
240
kDa
Cdc13-13xMyc
150
100
75
GAPDH

Figure S4. Top1-Myc expression from the YETI overexpression strain LP124 induced with 0, 2.5, or 5 nM estradiol as compared to the WT control (Border Strain LP55) (one-way ANOVA with Dunnett's test for multiple comparisons, **p=0.0027, ***p=0.0005, **p=0.0035 n=3 biological replicates). Tub1 is used as the loading control.

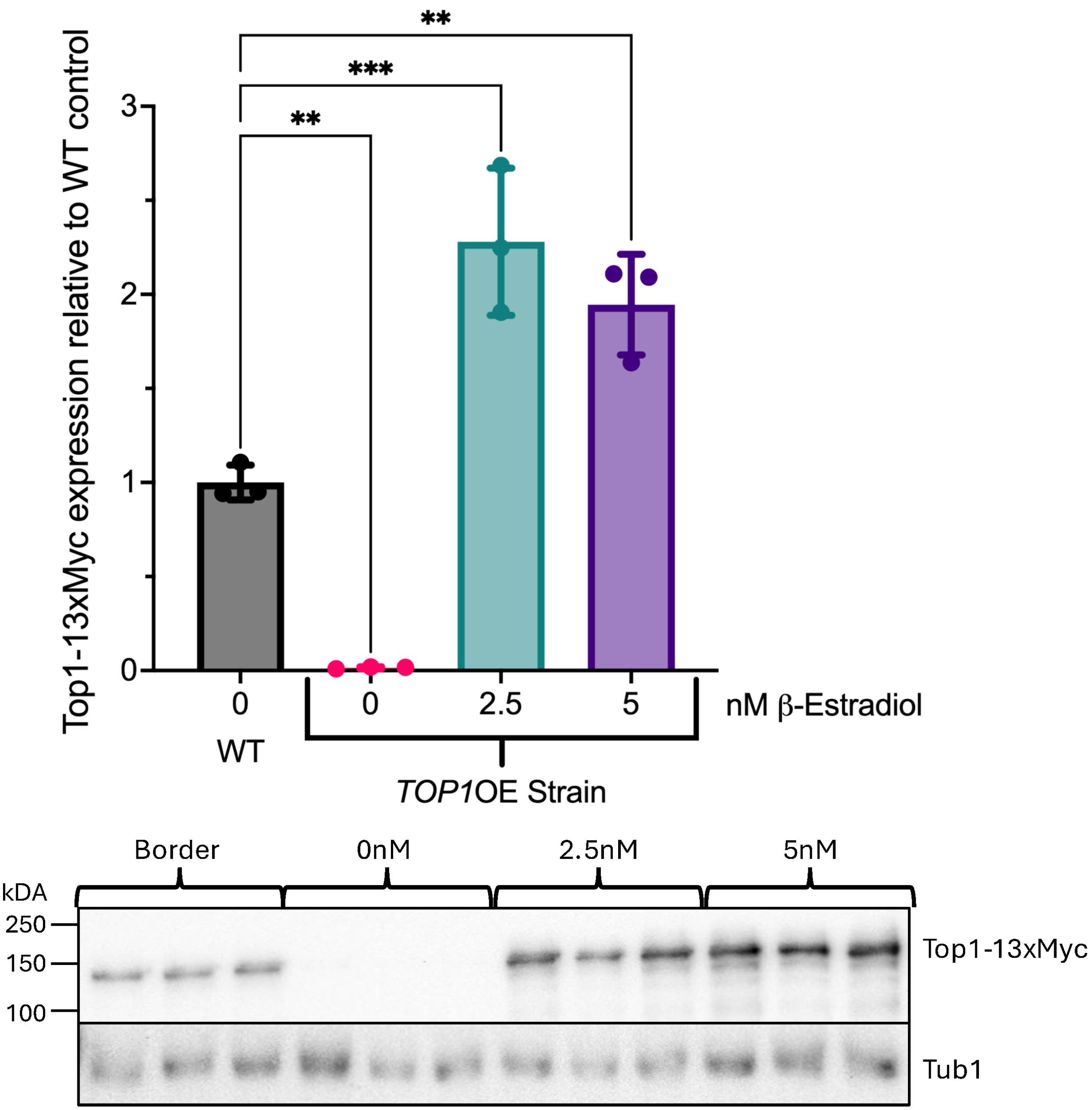

Figure S5. Verification by qRT-PCR of *TOP1* mRNA overexpression by low copy (CEN/ARS) or high copy (2µ) *LEU2* vectors expressing either wildtype (*TOP1*-WT) or catalytically dead (*top1*-CD) under control of the native *TOP1* promoter. Fold change is relative to a strain harboring the empty vector control. (CEN/ARS: **p=0.0014, **p=0.0037, one-way ANOVA with Dunnett's test for multiple-comparisons, n=3 biological replicates) (2µ: **p=0.0023, *p=0.0325 one-way ANOVA with Dunnett's test for multiple-comparisons, n=3 biological replicates).

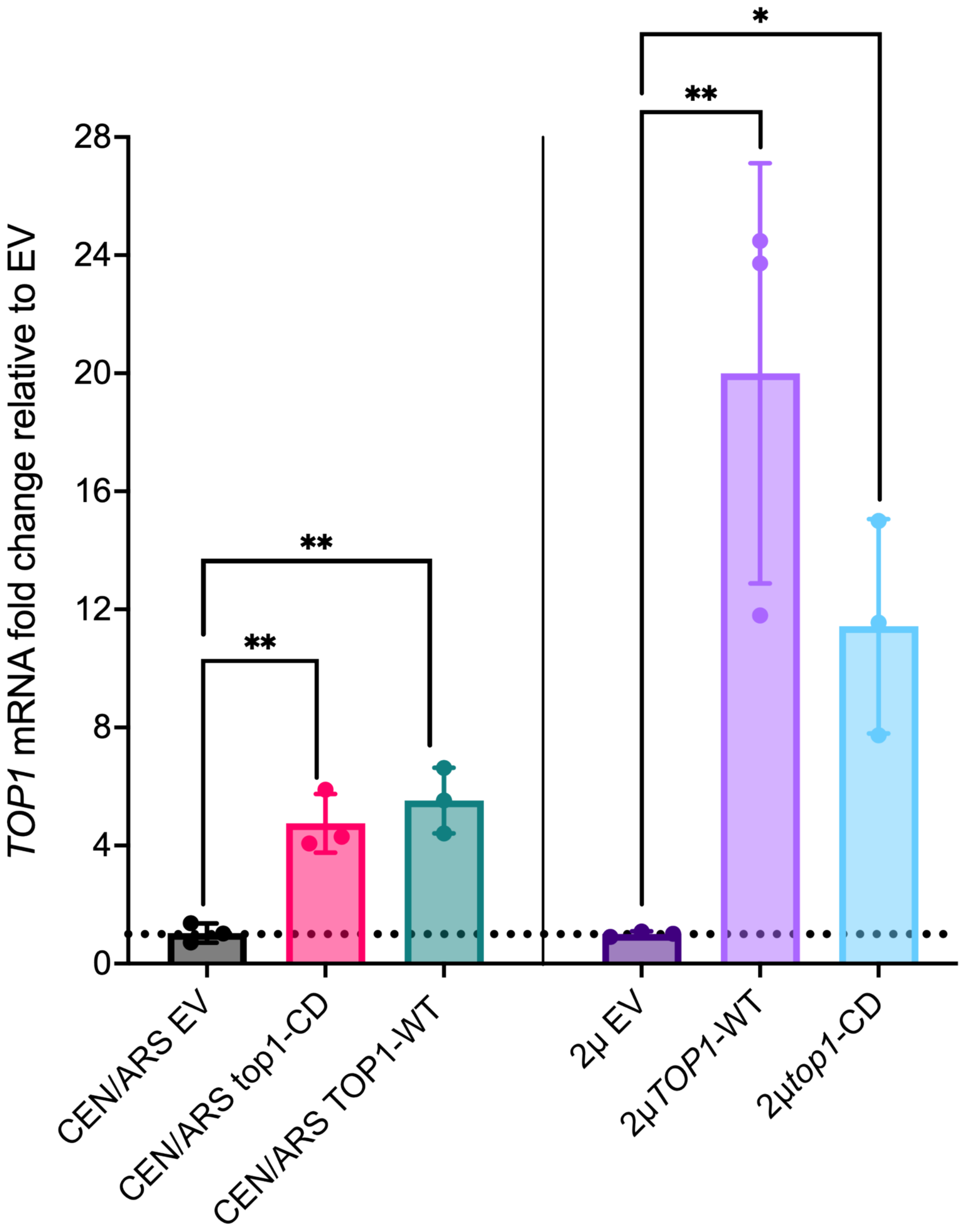

*
**
**
**
28
24
20
16
12
8
4
0
TOP1 mRNA fold change relative to EV
CEN/ARS EV
CEN/ARS *top1*-CD
CEN/ARS TOP1-WT
2µ EV
2µ*TOP1*-WT
2µ*top1*-CD

Figure S6. Quantitative rDNA silencing assays. Growth rates of the *mURA3* reporter strain YNM44 in liquid SC-Leu-Ura or SC-Leu+FOA media is normalized to the growth rate in SC-Leu media. The SC-Leu normalized growth rate for each treatment was normalized to the average growth rate of the empty vector control. Significant growth differences between the empty vector control and the *TOP1*-WT, *TOP1*-catalytically dead (*TOP1*-CD), *NET1*, or *SIR2* plasmids are calculated by ANOVA with Dunnett's test for multiple comparisons. (From left to right p = 0.059, 0.003**, 0.001***, 0.003** for the SC-Ura plot and p = 0.353, <0.001****, <0.001****, <0.001**** for the SC-FOA plot, n=3 biological replicates.)

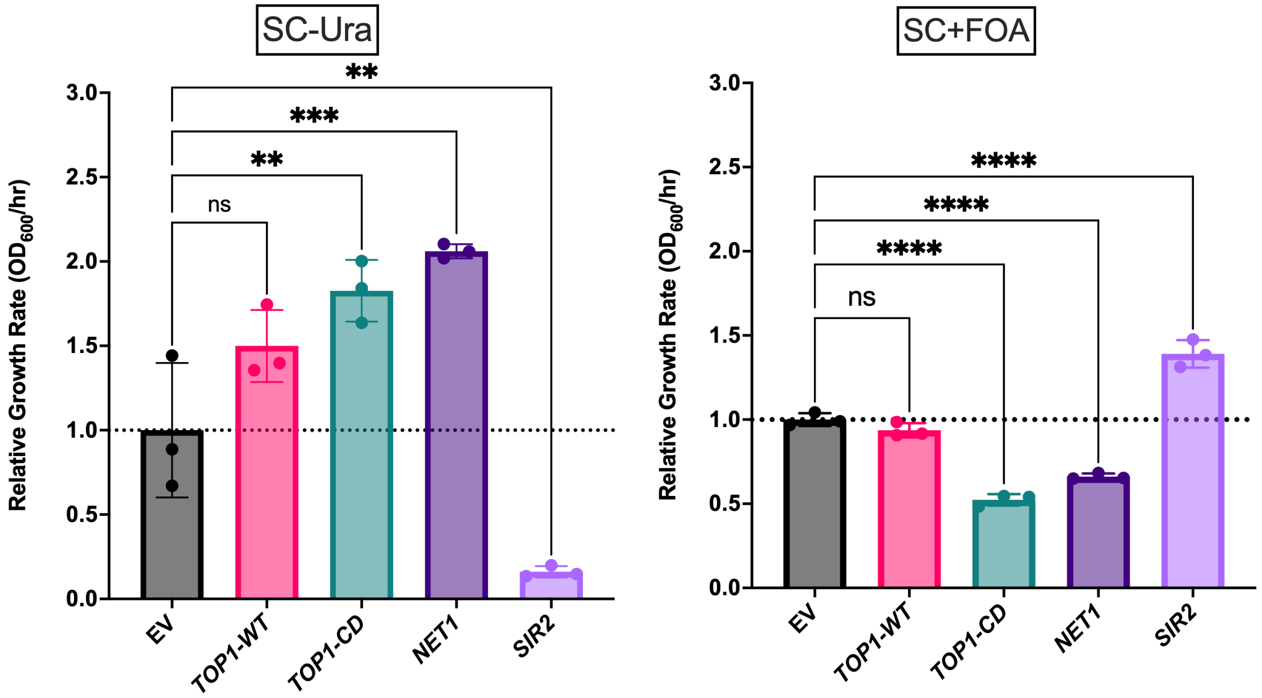

Table S1. List of yeast strains.

Strain	Genotype	Source
BY4743 (SY38)	*MAT a his3Δ1 leu2Δ0 LYS2 met15Δ0 ura3Δ0/ MATα his3Δ1 leu2Δ0 lys2Δ0 MET15 ura3Δ0*	Brachmann et al. 1999
BY4741 (SY41)	*MAT a his3Δ1 leu2Δ0 LYS2 met15Δ0 ura3Δ0*	Brachmann et al. 1999
YPH499 (SY104)	*MAT a ura3-52 lys2-801 ade2-101 trp1-Δ63 his3-Δ200 leu2-Δ1*	Sikorski et al. 1989
MD188	*MAT a his3Δ1 leu2Δ0 LYS2 met15Δ0 ura3Δ0 Hsp104-13xMyc::KanMX*	This study
YRH919	*MAT α his3Δ200 leu2Δ1 trp1Δ63 ura3-167 nts1Δ::URA3/HIS3 Top1-13xMyc::KanMX*	This study
LP128	*MAT a his3Δ1 leu2Δ0 LYS2 met15Δ0 ura3Δ0 Top1-13xMyc::KanMX*	This Study
LP55	*MAT a [HAP1::natMX::ACT1pr-Z3EV-ENO2term] ura3Δ0 can1Δ::STE2pr-Sphis5 his3Δ1 lyp1Δ*	Arita et al. 2021
LP56	*MAT a [barcode::URA3::Z3EVpr-Top1] [HAP1::natMX::ACT1pr-Z3EV-ENO2term] ura3Δ0 can1Δ::STE2pr-Sphis5 his3Δ1 lyp1Δ*	Arita et al. 2021
LP181	*MAT a ura352 lys2-801 ade2-101 trp1Δ63 his3Δ200 leu2Δ1 + pRS416*	This study
LP183	*MAT a ura352 lys2-801 ade2-101 trp1Δ63 his3Δ200 leu2Δ1 + pNK66 (YCpSCTOP1-U)*	This study
LP185	*MAT a ura352 lys2-801 ade2-101 trp1Δ63 his3Δ200 leu2Δ1 + pNK67 (YCpSCTOP1 Y727F-U)*	This study
LP257	*MAT a ura352 lys2-801 ade2-101 trp1Δ63 his3Δ200 leu2Δ1::SIR2-LEU2 + pRS416*	This study
LP263	*MAT a ura352 lys2-801 ade2-101 trp1Δ63 his3Δ200 leu2Δ1::SIR2-LEU2 + pNK66 (YCpSCTOP1-U)*	This study
LP269	*MAT a ura352 lys2-801 ade2-101 trp1Δ63 his3Δ200 leu2Δ1::SIR2-LEU2 + pNK67 (YCpSCTOP1 Y727F-U)*	This study
YNM44	*MAT α his3Δ200 leu2Δ1 ura3-167 RDN1 (Ter1-R)::mURA3-HIS3*	Buck et al. 2016
LP301	YNM44 *Δtop1::KanMX*	This study
AW1	YNM44 + pRS415	This study
AW2	YNM44 + pAW2	This study
AW3	YNM44 + pAW4	This study
AW4	YNM44 + pSB794	This study
AW5	YNM44 + pSB764	This study
AW6	YNM44 + pRS425	This study
AW7	YNM44 + pAW6	This study
AW8	YNM44 + pAW8	This study
AW9	YNM44 + pSB790	This study

AW10	YNM44 + pSB766	This study
AW11	LP301 + pRS415	This study
AW12	LP301 + pAW2	This study
AW13	LP301 + pAW4	This study
AW14	LP301 + pSB794	This study
AW15	LP301 + pSB764	This study
AW16	LP301 + pRS425	This study
AW17	LP301 + pAW6	This study
AW18	LP301 + pAW8	This study
AW19	LP301 + pSB790	This study
AW20	LP301 + pSB766	This study
AW41	JS124 + pRS415	This study
AW42	JS124 + pAW2	This study
AW43	JS124 + pAW4	This study
AW44	JS124 + pSB794	This study
AW45	JS124 + pSB764	This study
AW46	JS124 + pRS425	This study
AW47	JS124 + pAW6	This study
AW48	JS124 + pAW8	This study
AW49	JS124 + pSB790	This study
AW50	JS124 + pSB766	This study
MD207	YPH499 *SIR2-13xMyc::KanMX*	This study
MD208	MD207 + pRS416	This study
MD209	MD207 + pNK66 (*YCpSCTOP1-U*)	This study
MD210	MD207 + pNK67 (*YCpSCtop1Y727F-U*)	This study
SY533	BY4741 *sir2Δ::URA3*	This study
LP124	LP56 *TOP1-13xMyc::KanMX*	This Study
UCC6277 (RGY525)	*MATa ade2Δ::hisG his3Δ200 leu2Δ0 lys2Δ0 met15Δ0 trp1Δ63 ura3Δ0 ADE2-TEL-VR sir4::hphMX::pTDH3-1Myc-sir4-9::LEU2 CDC13::TRP1::cdc13-1-9Myc*	Gardner et al. 2005

Table S2. **List of plasmids**.

Plasmid	Description	Source
pNK66 (YCpSc*TOP1*-U)	WT *TOP1* under its native promoter in a pRS416 yeast CEN/ARS vector.	Colley et al. 2004 (Gifted by Nyun Kim.)
pNK67 (YCpSc*top1* Y727F-U)	Catalytically dead Top1 under its native promoter in pRS416 yeast CEN/ARS vector.	Colley et al. 2004 (Gifted by Nyun Kim.)
pAsc415	CEN/ARS *LEU2* (*Asc*I site ligated into *SmaI* site of pRS415)	Buck et al. 2002
pSB794	pAsc415-*SIR2*	Buck et al. 2002
pSB764	pAsc415-*NET1*	Buck et al. 2002
pAW4	pAsc415-*TOP1* (WT)	This Paper
pAW2	pAsc415-*top1*Y727F (CD)	This Paper
pAsc425	*2µ LEU2* (*Asc*I site ligated into *SmaI* site of pRS425)	Buck et al. 2002
pSB766	pAsc425-*SIR2*	Buck et al. 2002
pSB790	pAsc425-*NET1*	Buck et al. 2002
pAW6	pAsc425-*TOP1* (WT)	This Paper
pAW8	pAsc425-*top1*Y727F (CD)	This Paper

Table S3. **List of DNA oligos**.

Oligo	Description	Sequence 5'-3'
JS3878	*HSP104-13xMyc* Fw	CGATAATGAGGACAGTATGGAAATTGATGATGACC TAGATCGGATCCCCGGGTTAATTAA
JS3879	*HSP104-13xMyc* Rv	ATTCTTGTTCGAAAGTTTTAAAAATCACACTATAT TAAAGAATTCGAGCTCGTTTAAAC
JS3611	*TOP1-13xMyc* Fw	GTTCCGATTGAAAAGATTTT
JS3612	*TOP1-13xMyc* Rv	CTTCCTAGTAACCCTAATGC
JS3398	RTqPCR *TOP1* Fw	CGAGAAGAAGAAGAAGAGGAGG
JS3399	RTqPCR *TOP1* Rv	TGGTAAGGGCTGGTATGGTG
JS3976	RTqPCR *UBC6* Fw	ATTGGATGAGGGGGATGCGGCA
JS3977	RTqPCR *UBC6* Rv	AGCGCGTATTCTGTCTTCAGGGT
JS4054	*TOP1* short gene PCR cloning (AscI) Fw	TAGGCGCGCCATATGATCGATGCACGTAAAGAAC
JS4055	*TOP1* short gene PCR cloning (AscI) Rv	TAGGCGCGCCAAGAGATACGGACAATACGTTTC
JS1191	*SIR2-13xMyc* Fw	CGTGTATGTCGTTACATCAGATGAACATCCCAAAA CCCTCCGGATCCCCGGGTTAATTAA
JS1192	*SIR2-13xMyc* Rv	TATTAATTTGGCACTTTTAAATTATTAAATTGCCTT CTACGAATTCGAGCTCGTTTAAAC

Table S4. CHX Chase Assay P-Value reporting.

Dunnett's multiple comparisons test	Mean Diff.	95.00% CI of diff.	Below threshold?	Summary	Adjusted P Value
**Figure 2C**					
0 vs. 30	0.2355	-0.3085 to 0.7795	No	ns	0.4948
0 vs. 60	0.09269	-0.4513 to 0.6367	No	ns	0.9264
0 vs. 90	0.3247	-0.2193 to 0.8687	No	ns	0.2712
**Figure 2D**					
0 vs. 1	-0.1274	-1.165 to 0.9098	No	ns	0.9694
0 vs. 2	-0.219	-1.256 to 0.8182	No	ns	0.8746
0 vs. 4	-0.01247	-1.050 to 1.025	No	ns	>0.9999
**Figure 2E (0-2 buds)**					
0 vs. 30	-0.0736	-0.6281 to 0.4809	No	ns	0.9622
0 vs. 60	-0.0304	-0.5849 to 0.5241	No	ns	0.997
0 vs. 90	0.09023	-0.4643 to 0.6448	No	ns	0.9347
**Figure 2E (6-7 buds)**					
0 vs. 30	-0.2456	-1.131 to 0.6397	No	ns	0.7688
0 vs. 60	-0.0722	-0.9575 to 0.8131	No	ns	0.9904
0 vs. 90	-0.03633	-0.9216 to 0.8489	No	ns	0.9987

Supplementary References

Arita, Y., Kim, G., Li, Z., Friesen, H., Turco, G., Wang, R. Y., Climie, D., Usaj, M., Hotz, M., Stoops, E. H., Baryshnikova, A., Boone, C., Botstein, D., Andrews, B. J., and McIsaac, R. S. (2021) A genome-scale yeast library with inducible expression of individual genes. *Mol Sys Biol.* **17**, e10207.

Brachmann, C. B., Davies, A., Cost, G. J., Caputo, E., Li, J., Hieter, P., and Boeke, J. D. (1998) Designer deletion strains derived from *Saccharomyces cerevisiae* S288C: A useful set of strains and plasmids for PCR-mediated gene disruption and other applications. *Yeast*. **14**, 115–132.

Buck, S. W., Sandmeier, J. J., and Smith, J. S. (2002) RNA polymerase I Propagates unidirectional spreading of rDNA silent chromatin. *Cell*. **111**, 1003–1014.

Buck, S. W., Maqani, N., Matecic, M., Hontz, R. D., Fine, R. D., Li, M., and Smith, J. S. (2016) RNA Polymerase I and Fob1 contributions to transcriptional silencing at the yeast rDNA locus. *Nucleic Acids Res*. **44**, 6173–6184.

Colley, W. C., van der Merwe, M., Vance, J. R., Burgin, A. B., Jr., and Bjornsti, M.-A. (2004) Substitution of conserved residues within the active site alters the cleavage religation Equilibrium of DNA Topoisomerase I. *J Biol Chem*. **279**, 54069–54078.

Gardner, R. G., Nelson, Z. W., and Gottschling, D. E. (2005) Degradation-mediated protein quality control in the nucleus. *Cell*. **120**, 803–815.

Sikorski, R. S., and Hieter, P. (1989) A system of shuttle vectors and yeast host strains designed for efficient manipulation of DNA in *Saccharomyces cerevisiae*. *Genetics*. **122**, 19–27.
